# Intestinal Metagenomes and Metabolomes in Healthy Young Males: Inactivity and Hypoxia Generated Negative Physiological Symptoms Precede Microbial Dysbiosis

**DOI:** 10.3389/fphys.2018.00198

**Published:** 2018-03-13

**Authors:** Robert Šket, Tadej Debevec, Susanne Kublik, Michael Schloter, Anne Schoeller, Boštjan Murovec, Katarina Vogel Mikuš, Damjan Makuc, Klemen Pečnik, Janez Plavec, Igor B. Mekjavić, Ola Eiken, Zala Prevoršek, Blaž Stres

**Affiliations:** ^1^Group for Microbiology and Microbial Biotechnology, Department of Animal Science, Biotechnical Faculty, University of Ljubljana, Ljubljana, Slovenia; ^2^Department of Automation, Biocybernetics and Robotics, Jozef Stefan Institute, Ljubljana, Slovenia; ^3^Faculty of Sport, University of Ljubljana, Ljubljana, Slovenia; ^4^Research Unit for Comparative Microbiome Analysis, Helmholtz Zentrum München - German Research Center for Environmental Health, Neuherberg, Germany; ^5^Machine Vision Laboratory, Faculty of Electrical Engineering, University of Ljubljana, Ljubljana, Slovenia; ^6^Department of Biology, Biotechnical Faculty, University of Ljubljana, Ljubljana, Slovenia; ^7^Slovenian NMR Centre, National Institute of Chemistry, Ljubljana, Slovenia; ^8^Department of Environmental Physiology, Swedish Aerospace Physiology Centre, Royal Institute of Technology, Stockholm, Sweden; ^9^Group for Genetics, Animal Biotechnology and Immunology, Department of Animal Science, Biotechnical Faculty, University of Ljubljana, Ljubljana, Slovenia; ^10^Center for Clinical Neurophysiology, Faculty of Medicine, University of Ljubljana, Ljubljana, Slovenia

**Keywords:** metagenome, metabolome, human gut, microbiome, dysbiosis, exercise, inactivity, hypoxia

## Abstract

We explored the metagenomic, metabolomic and trace metal makeup of intestinal microbiota and environment in healthy male participants during the run-in (5 day) and the following three 21-day interventions: normoxic bedrest (NBR), hypoxic bedrest (HBR) and hypoxic ambulation (HAmb) which were carried out within a controlled laboratory environment (circadian rhythm, fluid and dietary intakes, microbial bioburden, oxygen level, exercise). The fraction of inspired O_2_ (F_i_O_2_) and partial pressure of inspired O_2_ (P_i_O_2_) were 0.209 and 133.1 ± 0.3 mmHg for the NBR and 0.141 ± 0.004 and 90.0 ± 0.4 mmHg (~4,000 m simulated altitude) for HBR and HAmb interventions, respectively. Shotgun metagenomes were analyzed at various taxonomic and functional levels, ^1^H- and ^13^C -metabolomes were processed using standard quantitative and human expert approaches, whereas metals were assessed using X-ray fluorescence spectrometry. Inactivity and hypoxia resulted in a significant increase in the genus *Bacteroides* in HBR, in genes coding for proteins involved in iron acquisition and metabolism, cell wall, capsule, virulence, defense and mucin degradation, such as beta-galactosidase (EC3.2.1.23), α-L-fucosidase (EC3.2.1.51), Sialidase (EC3.2.1.18), and α-N-acetylglucosaminidase (EC3.2.1.50). In contrast, the microbial metabolomes, intestinal element and metal profiles, the diversity of bacterial, archaeal and fungal microbial communities were not significantly affected. The observed progressive decrease in defecation frequency and concomitant increase in the electrical conductivity (EC) preceded or took place in absence of significant changes at the taxonomic, functional gene, metabolome and intestinal metal profile levels. The fact that the genus *Bacteroides* and proteins involved in iron acquisition and metabolism, cell wall, capsule, virulence and mucin degradation were enriched at the end of HBR suggest that both constipation and EC decreased intestinal metal availability leading to modified expression of co-regulated genes in *Bacteroides* genomes. Bayesian network analysis was used to derive the first hierarchical model of initial inactivity mediated deconditioning steps over time. The PlanHab wash-out period corresponded to a profound life-style change (i.e., reintroduction of exercise) that resulted in stepwise amelioration of the negative physiological symptoms, indicating that exercise apparently prevented the crosstalk between the microbial physiology, mucin degradation and proinflammatory immune activities in the host.

## Introduction

Genetic and environmental factors involved in the onset of obesity result in a complex process often associated with the development of several chronic medical conditions (i.e., insulin resistance, hyperglycemia, hypertriglycemia, dyslipidemia, hypertension; Boulangé et al., 2016) and the activation of the immune system (Gregor and Hotamisligil, 2011). In healthy individuals, insulin was shown to elicit glucose uptake in peripheral organs and the use of extracellular glucose by the body resulting in increased tissue glycolysis and respiration, accumulation of glucose and lipids by stimulation of glycogenesis and lipogenesis, thereby also supporting protein synthesis (Saltiel and Kahn, 2001; Perry et al., 2014; Boulangé et al., 2016). On the other hand, impaired insulin action in peripheral organs results in a loss of sensitivity to insulin, termed insulin resistance that triggers fasting hyperglycemia, increased hepatic lipid synthesis, dyslipidemia, hypertension, cumulating in fat accumulation and increased adiposity in the long run, that are all associated with higher immune system activity levels (Boulangé et al., 2016).

Excessive calorie intake leads to saturation of the system with high-energy value molecules giving rise to increased fat accumulation that activates the production of effector molecules (cytokines) and cells primarily involved in innate immunity (Gregor and Hotamisligil, 2011; Boulangé et al., 2016). This production results in a chronic, low-grade inflammatory status, gives rise to recruitment and activation of many different types of mature immune cells in different tissues that cumulatively further modify the tissue environment and reinforce the inflammatory process (Lumeng and Saltiel, 2011; Sell et al., 2012; Boulangé et al., 2016).

Most studies thus far focused on the beneficial effects of re-introduction of exercise to obese population to alleviate these negative effects on one side, and to understand the effects of athletic overtraining on the other (Clarke et al., 2014; Barton et al., 2017). Recent studies clearly illustrated how exercise, microbiota and its metabolic functions are mutually dependent and responsive to physiological variations due to exercise (Clarke et al., 2014; Barton et al., 2017; Monda et al., 2017; Šket et al., 2017a,b). However, there is an obvious lack of data on the initial changes in human microbiome and physiology during acute cessation of exercise.

In order to improve our understanding of consequences of acute and prolonged inactivity and hypoxia on human pathophysiology the PlanHab experimental setup was adopted (Debevec et al., 2014; Simpson et al., 2016; Šket et al., 2017a,b). The PlanHab experiment was conducted according to European Space Agency (ESA) and NASA core bedrest data collection SOP (Standardization of bed rest study conditions 1.5, August 2009), included controlled daily water and nutritional intake in addition to controlled atmospheric oxygen content, circadian rhythm, microbial ambiental burden and 24/7 medical surveillance (Debevec et al., 2014; Simpson et al., 2016; Šket et al., 2017a,b).

**Figure 1:**
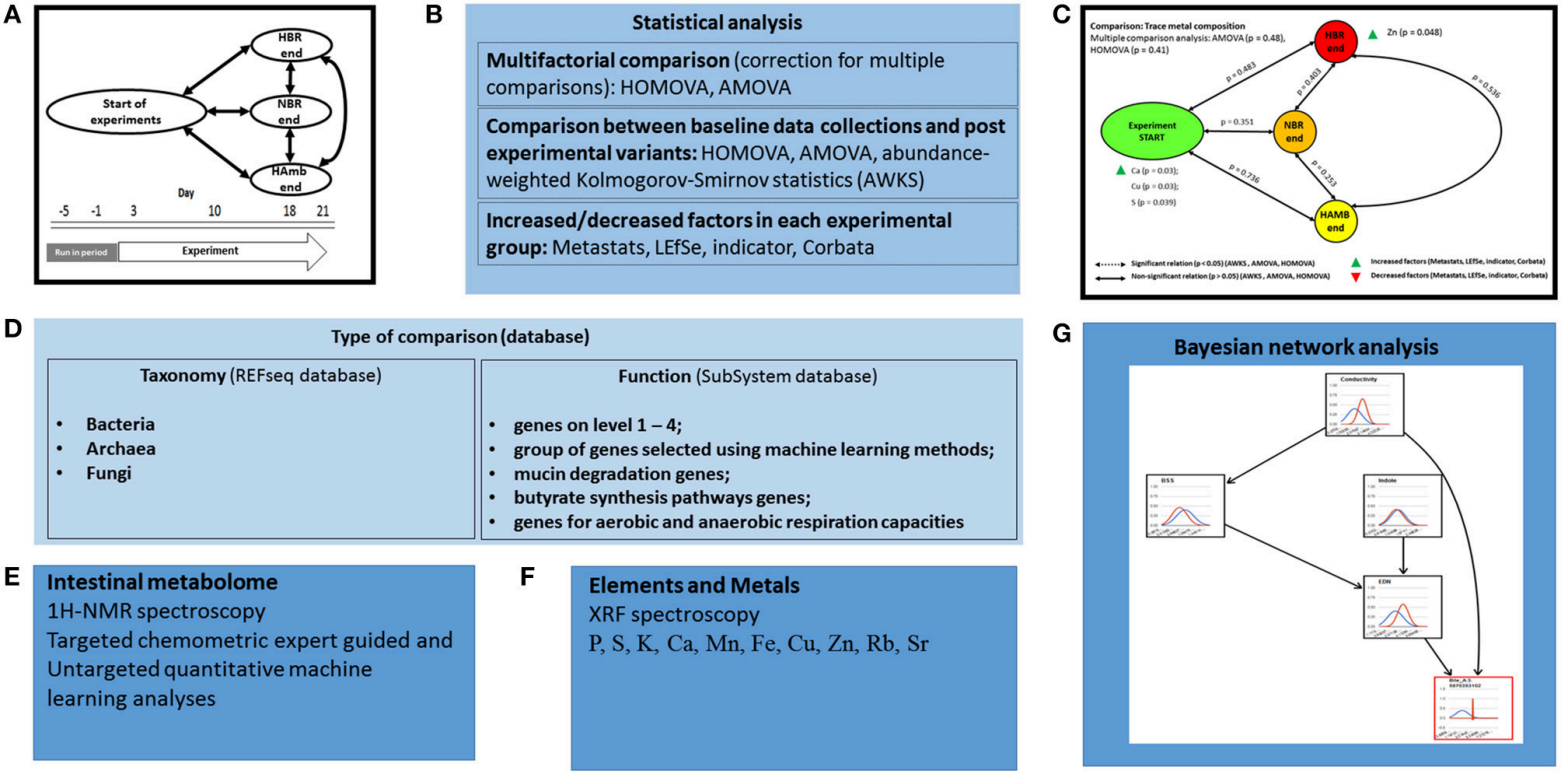
Schematic outline of the PlanHab experiments and analysis workflow described in this study: general outline of NBR, HBR and HAmb sampling and analyses **(A)**; **(B)** overview of the statistical analyses; **(C)** example of results; **(D)** taxonomic and functional metagenome annotation; **(E)** analysis of intestinal metabolome; **(F)** analysis of intestinal elements and metals; **(G)** integrative analysis and modeling using Bayesian network analysis.

In this study, multifaceted nature of the intestinal microbiota of healthy male test participants was explored during the 5-day run-in and three consecutive experimental phases [21-day normoxic bed rest (NBR), hypoxic bedrest (HBR) and hypoxic ambulation (HAmb); (Šket et al., 2017a,b)]. A number of variables linked to the interplay between intestinal microbiome and intestinal environment were measured: (i) Shot-gun metagenomes of intestinal microbial communities at taxonomic and functional levels, (ii) intestinal nuclear magnetic resonance (^1^H-NMR and ^13^C-NMR) metabolomes; (iii) X-ray fluorescence (XRF) spectroscopy of elements, and (iv) Bayesian network analysis of significantly different intestinal parameters and metabolites (Figure 1).

A number of negative physiological symptoms related to obesity and metabolic syndrome have been observed to a large extent in the PlanHab study in a dose dependent manner over the course of 21-day experimental period in HBR and NBR, but were absent from HAmb variant (Debevec et al., 2014, 2016; Rittweger et al., 2016; Simpson et al., 2016; Stavrou et al., 2016; Šket et al., 2017a,b; Strewe et al., 2017). In addition, the observed negative symptoms faded effectively in 14, 10, <4 days for HBR, NBR, and HAmb, respectively (Debevec et al., 2014; Šket et al., 2017a,b). Many of the microbial parameters such as butyrate producing microbial community, the general bacterial and archaeal microbial community were shown to lag behind the changes in human physiology and intestinal environment (Šket et al., 2017a,b), suggesting a time-dependent and complex interplay between the host physiology (including apparent constipation), immunity (inflammation), controlled diet, intestinal environment variables and microbiome physiology during the acute cessation of exercise. First analyses suggested that shifts in intestinal environment gave rise to modified microbial activity and metabolism toward degradation of host mucus layer in bedrest variants (HBR, NBR) on one side, and the production of beneficial indole derivatives in healthy HAmb on the other (Figure S1).

We hypothesized that an acute reduction in physical activity (complete inactivity) would result in (i) notable changes in the taxonomic and functional composition of intestinal tract metagenome; (ii) increased abundance of functional gene categories linked to elevated levels of the genus *Bacteroides* in the HBR variant reported before (Šket et al., 2017b); (iii) distinct intestinal metabolomes; and (iv) modified levels of various elements and trace metals in the intestine environment; providing sufficient data to establish (v) a Bayesian model linking relevant parameters and enabling the forecast of metabolic states. As an additional confounding factor aggravating the observed physiological changes within the 21-day experiment, the systemic hypoxia during inactivity was expected to point to the role of mitochondrial stress whereas ambulation in hypoxia was anticipated to at least partially ameliorate these inflammatory effects due to the increased physical activity levels, established hydrostatic pressures (Debevec et al., 2014; Šket et al., 2017a,b) and maintained posture-related muscle activity (Miles-Chan and Dulloo, 2017).

## Materials and methods

### Experimental setup

Experimental setup of the PlanHab study was extensively detailed before (Debevec et al., 2014, 2016; Rittweger et al., 2016; Simpson et al., 2016; Stavrou et al., 2016; Šket et al., 2017a,b; Strewe et al., 2017). For detailed experimental protocols, please see Šket et al. (2017a,b). In essence, the PlanHab study was carried out between October 2012 and December 2013 at the “Hypoxic Facility” of the Olympic Sport Center Planica in Rateče, Slovenia, according to the European Space Agency's standardization plan for bed rest studies (ESA, 2009). For this study, each participant underwent 5 days of baseline data collection during which participants were ambulant, 21 intervention days and 5–14 days of medical follow-up (Debevec et al., 2014; Šket et al., 2017a). For this study 11 healthy men with a mean age of mean age (±SD) of 26.4 ± 4.6 years, a height of 179.6 ± 4.7 cm, a mass of 75.9 ± 9.3 kg and a body mass index of 23.5 ± 2.7 kg/m^2^ were enrolled as described before (Debevec et al., 2014; Šket et al., 2017a,b).

### Microbial metagenome sequencing

The extracted genomic DNA (Šket et al., 2017a,b) was used as a template for whole shotgun sequencing. Approximately 200 ng of microbial DNA was used for shearing of the long DNA fragments with Covaris ultrasonicator. Quantity and quality of DNA were examined using DNA Agilent Bioanalyzer 2,100 and Quant-iT™ PicoGreen DNA Fragment Analyzer reagents DNF-473 Standard Sensitivity NGS Fragment Analysis Kit.

Metagenomic libraries were constructed using NEBNext Ultra DNA Library Prep Kit (Illumina Inc., USA) according to the manufacturer's protocol. First end repair of sonicated DNA and ligation of adapters was performed. Then cleaning step and size selection procedure of adapter ligated DNA were performed using magnetic beads. After that step only fragments of DNA around 650 base pairs long remained for downstream reactions.

Furthermore, indexing PCR and amplification of adapter ligated DNA was performed, followed by another cleaning step with magnetic beads. Prepared libraries were diluted to 4 nM, pooled equimolar and shotgun sequenced using Illumina MiSeq platform (Illumina Inc., USA). Sequencing run produced 1.8 × 10^7^ reads in total, 1 × 10^6^ ± 1.9 × 10^5^ (mean ± SD) per sample.

### Bioinformatic and statistical analysis of microbial metagenome

Taxonomical and functional annotation of metagenomic datasets was performed using Metagenomic Rapid Annotations using Subsystems Technology (MG-Rast server) (Meyer et al., 2008). The data was compared to REFSeq database for taxonomical annotation and Subsystems database for functional annotation using a maximum e-value of 1e^−5^, a minimum identity of 60%, and a minimum alignment length of 15 base pairs and amino acids respectively.

To analyze relationship between start vs. end of each variant, and between ends of particular variants, a number of established approaches was used: parsimony, weighted UniFrac, uweighted UniFrac, AMOVA, HOMOVA, lefse, indicator and metastats tests with 999 permutations were used as implemented in mothur (Schloss et al., 2009). Multiple-group comparisons were performed using Benjamini-Hochberg false discovery rate (FDR) multiple test correction (Benjamini and Hochberg, 1995; Benjamini and Yekutieli, 2001; Šket et al., 2017a,b). In addition, a test statistic Abundance-Weighted Kolmogorov-Smirnov (AWKS) as implemented in Corbata (CORe microBiome Analysis Tools; Li et al., 2013; Šket et al., 2017b) was used for calculations of the difference between variants at the taxonomic or functional profiles. The congruency of results from the various tests deployed was schematically summarized (Figure 1) and taken to reflect the signal observed at various levels of microbiome data integrating over the underlying statistical assumptions of particular separate analytical approach adopted in this study. The use of a concerted approach of various statistical approaches enabled us to detect the most evident congruent changes in microbiome.

### Intestinal metabolome analysis using proton nuclear magnetic resonance (^1^H-NMR)

Fecal samples (200 mg of dry matter) were resuspended in 800 μL of NMR phosphate buffer and centrifuged at 10,000 g for 30 min at 4°C to remove fine particles. Samples were filtered through 0.22 μm HPLC compatible filters (Millipore, Germany), 400 μL aliquots were mixed with 200 uL ^1^H-NMR buffer as described before (Beckonert et al., 2007) and stored at −25°C until analysis. Phosphate buffer (pH 7.4) was prepared by weighing 1.443 g NaH_2_PO_4_, 0.263 g NaH_2_PO_4_, 2 mM TSP, and 1 mM NaN3 into 50 mL volumetric flask. Ten milliliter of D_2_O was added and filled up to 50 mL with Milli-Q water. Before analysis, samples were thawed at room temperature, centrifuged at 12,000 g for 5 min at 4°C. In total, 550 μL of each sample was transferred into 5 mm NMR tube.

^1^H-NMR spectra were acquired on an Agilent Technologies DD2 600 MHz NMR spectrometer equipped with 5 mm HCN Cold probe. The 2D experiments were measured on Agilent Technologies (Varian) VNMRS 800 MHz NMR spectrometer equipped with 5 mm HCN Cold probe. All experiments were measured at 25°C. ^1^H-NMR spectra of the samples were recorded with spectral width of 9.0 kHz, relaxation delay 2.0 s, 32 scans and 32 K data points. Water signal was suppressed using Double-pulsed field gradient spin echo (DPFGSE) pulse sequence. Heteronuclear single quantum coherence spectrum (HSQC) was acquired with spectral widths of 9.0 kHz and 40 kHz for ^1^H- and ^13^C-dimensions, respectively, 1,536 complex points for ^1^H-dimension, relaxation delay 1.5 s, 160 number of transients and 128 time increments. Total correlated spectrum (TOCSY) was measured with ^1^H spectral widths of 7.0 kHz, 4096 complex points, relaxation delay 1.5 s, 32 number of transients and 144 time increments. The ^1^H and 2D spectra were apodized with an exponential function and a cosine-squared function, respectively, and zero filled before Fourier transform. All NMR spectra were processed and analyzed using VNMRJ (Agilent/Varian) and Sparky (UCSF) software and MestReNova.

The resulting spectra were consequently analyzed in two complementary ways: (i) human expert chemometric untargeted metabolomics, including 2D spectra, and (ii) targeted quantitative metabolomics using Chenomx NMR Suite version 8.3 (Chenomx, Canada). For the latter, all spectra were randomly ordered for spectral fitting using ChenomX profiler. Metabolites analyzed in this study were thus independently identified using 2D NMR spectroscopy (see Supplementary Material, Figure S2 and Table S1) and with the support of Chenomx Compound Library extended by Human Metabolome Data Base (Wishart et al., 2013) the latter giving access to the use of chemical shift profiles of 674 compounds.

Three different approaches to asymmetric sparse matrix data analysis were adopted (Legendre and Legendre, 2012). Each compound concentration obtained was (i) normalized by dividing the measured concentration by the concentration of all metabolites in that sample; (ii) Box-Cox or log2 transformed; (iii) corrected for the total dissolved organic matter concentration (TSOC), determined as described before (Kolbl et al., 2017).

The significance of difference in the metabolic characteristics of various groups of samples was tested using ANOSIM, NP-MANOVA and expressed as an overlap in non-metric multidimensional scaling (nm-MDS) trait space (Gower and Euclidean distance measures). Stress function was used to select the dimensionality reduction whereas Shepard's plots were used to describe the correspondence between target and obtained ranks (Murovec et al., 2015). Benjamini Hochberg significance correction for multiple comparisons was used as described before (Šket et al., 2017a,b). The Methane Yield Database was used to estimate the methane potential of constituents of the Western-diet used within the PlanHab experiment (Šket et al., 2017a,b).

### X-ray fluorescence spectrometry (XRF) of intestinal metal content

Sample (10 g) was dried at 60°C, homogenized and 100 mg of subsamples were compressed using a pellet die and hydraulic press (Nečemer et al., 2008). Element analysis was performed by X-ray florescence spectrometry focusing on the following 10 elements: P, S, K, Ca, Mn, Fe, Cu, Zn, Rb, and Sr (Figure S3). XRF spectrometer based on Rh anode (35 kV) with 5 mm beam was used to irradiate the samples. XRF signal was detected with silicon drift diode (SDD) (Amptek). Spectra were analyzed in LabView and quantitative analysis was performed as described before (Kump et al., 1996). Dark matrix (the non-responsive elements) were determined by emission transmission method (Nečemer et al., 2008). Quality assurance for the element analysis was performed using standard reference materials: NIST SRM 1573a (tomato leaves, homogenized powder); CRM 129 (hay powder); and OU-10 (geological sample of Longmyndian greywacke, GeoPT24) (Likar et al., 2015). All statistical analyses were performed as described above.

### Bayesian network modeling

To explore the relationships between the immune, intestinal and environmental parameters associated significantly with the distribution of microbial communities in experimental variants over time observed before (Šket et al., 2017a,b) and in this study, Bayesian network analysis was conducted using the C++ utility (Ziebarth et al., 2013). A number of parameters (*n* = 261) was monitored over the course of the PlanHab experiment and a database containing these data was interrogated (Šket et al., 2017b). Only the intestinal parameters that differed significantly over the course of the PlanHab experiment were used (Figure S1). First, the structure of a network model that best explains the data was learned, and second, the model was used to make predictions about the interactions between the variables in the model. Global structure learning settings included (i) Maximum number of parents (*n* = 4) as the number of immediate parents for every node in the network to increase the speed of structure learning for larger networks and to avoid over-fitting a network model; (ii) Number of high-scoring networks to include in model averaging (*k* = 100) to increase the performance of the structure learning search; (iii) Model averaging selection threshold of directed edges to be included in the network given their posterior probabilities after model averaging (Ziebarth et al., 2013). All directed edges with posterior probabilities greater than the threshold were included in the network (*t* = 0.8); (iv) Number of tiers in the network which can then be used to specify structure learning constraints was not assigned in order to perform the structure learning from the data (Ziebarth et al., 2013).

The significant parameters identified in this and our recent studies (Šket et al., 2017a,b) were inspected and segmented according to the recorded time of significant deviation. The arranged data were superimposed next to the calculated Bayes network in order to check for the correspondence between the experimentally observed and calculated hierarchy governed by time frame of appearance.

## Results

### Taxonomic annotation of bacteria, archaea, and fungi

Altogether, 2.68 × 10^7^ taxonomic hits were assigned of which 2.66 × 10^7^ hits belonged to Bacteria. The most abundant bacterial phyla were Firmicutes (59.6 ± 13.3%) and Bacteroidetes (29.7 ±14.2%), followed by Actinobacteria (3.87 ± 2.02%), Proteobacteria (3.80 ± 1.27%), Fusobacteria (0.52 ± 0.12%) and Spirochaetes (0.48 ± 0.14%), in line with the general assembly of human microbiome (Candela et al., 2012) and our past observations on the same samples (Šket et al., 2017b).

**Figure 2:**
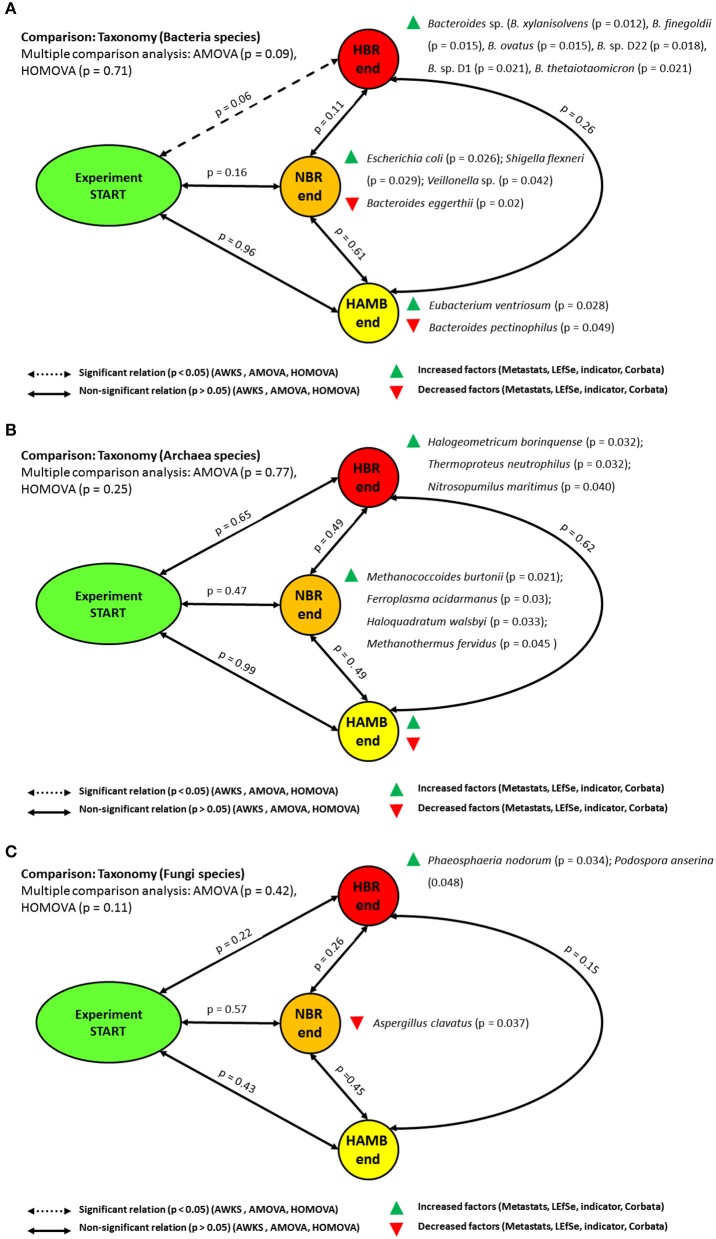
Schematic representation of the significant changes in the taxonomic annotation of metagenomes at the level of bacterial **(A)**, archaeal **(B)**, and fungal **(C)** microbial communities.

No significant differences were observed between initial and endpoint bacterial microbiomes at the level of bacterial taxonomic distribution using parsimony or unweighted Unifrac metrics based either on Bray-Curtis, ThetaYC and Jaccard indices. The group centroids (AMOVA) and dispersion (HOMOVA) between initial and endpoint experimental variants were at the margin of significance (*p* = 0.07) after the correction for multiple comparisons. Lefse, indicator species and metastats tests showed that *Eubacterium ventriosum* (*p* = 0.028), *Bacteroides* sp. (*p* = 0.045) and *Proteobacteria* [*Escherichia coli* (*p* = 0.026), *Shigella* sp. (*p* = 0.029), *Veillonella* sp. (*p* = 0.042)] were significantly enriched at the end of HAmb, HBR and NBR variants, respectively (Figure 2).

The members of *Eubacterium* that were enriched at the end of HAmb are intensively involved in anaerobic degradation of various plant polyphenols, e.g., flavonoids, through deconjugation of various sugars obtained through diet into derivatives with important physiological bioactivity for the host (Schneider and Blaut, 2000; Rowland et al., 2017). However, several studies have shown that the complete metabolism of polyphenol glycosides requires active involvement of complex intestinal microbial communities (Rowland et al., 2017).

Closer examination showed that the members of the genus *Bacteroides* were significantly enriched at the end of HBR variant [*B. xylanisolvens* (p = 0.012), *B. finegoldii* (*p* = 0.015), *B. ovatus* (*p* = 0.015), *B*. sp. D22 (*p* = 0.018); *B*. sp. D1 (*p* = 0.021), *B. thetaiotaomicron* (*p* = 0.021), *B. fragilis* (*p* = 0.033), *B. caccae* (*p* = 0.036), *B. cellulosilyticus* (*p* = 0.04), *B. capillosus* (*p* = 0.045), *B. dorei* (*p* = 0.048)]. In addition, *B. eggerthii* (*p* = 0.02) and *B. pectinophilus* (*p* = 0.049) were decreased at the end of NBR and HAmb variants. The results presented in this study are in line with our past analyses at the level of 16S rRNA amplicon sequencing (Šket et al., 2017b) that identified a significant increase in the presence of the members of the genus *Bacteroides* in HBR participants that belonged to species, previously linked to various dysbioses in humans (e.g., intestinal tract inflammation, obesity, insulin resistance, hyperglycemia, metabolic syndrome, T2D (Šket et al., 2017b). The elevated levels of the members of the genus *Bacteroides* were associated with a poor postprandial glucose response in HBR and NBR participants and other negative physiological symptoms (Simpson et al., 2016; Šket et al., 2017b). However, not all members of the genus *Bacteroides* interacted with their host in the same way, showing that different but highly related species can have very different effects on host metabolism, further suggesting that the key effectors of improved metabolism of the host in HAmb could be microbial metabolome, proteome and lipidome (Johnson et al., 2016).

On the other hand, the increase in *Proteobacteria* in NBR falls in line with past observations of potential metabolic endotoxemia (Kamada et al., 2013). Altered intestinal conditions were recently shown to facilitate the overgrowth of potentially harmful constituents of indigenous intestinal bacteria as some pathogens more efficiently utilize common resources under novel conditions established due to physical inactivity (Kamada et al., 2013). Another important strategy utilized by bacterial pathogens to further acquire a growth advantage over bacterial commensals is to stimulate host intestinal inflammation that additionally hinders commensal survival, but increases competitive advantages of pathogenic behavior in terms of nutrient acquisition (Kamada et al., 2013).

Archaeal diversity and composition were also assessed in the same samples (Figure 2). The majority of all archaeal hits (1.3 × 10^5^ sequences) was mostly assigned to phylum *Euryarchaeota* (93.4 ± 1.01%), followed by *Crenarchaeota* (5.46 ± 0.78%), *Thaumarchaeota* (0.54 ± 0.29%), *Korarchaeota* (0.53 ± 0.19%) and *Nanoarchaeota* (0.04 ± 0.06%). No significant differences in the taxonomic distribution at various levels of Archaea were identified using all of the above statistical tests between initial and end-point samples between experimental variants. These results are in-line with past observations of the significant difference in the microbial community of *Archaea* between the obese and healthy control groups (Pimentel et al., 2012; Bojanova and Bordenstein, 2016). However, archaeal methane production in humans has been epidemiologically and clinically associated with constipation related diseases (Triantafyllou et al., 2014), a trait observed in NBR and HBR variants of PlanHab but not HAmb (Šket et al., 2017a). Methane producers also had greater serum glucose content than non-methane subjects suggesting impaired glucose tolerance and higher susceptibility to hyperglycemia when challenged (Mathur et al., 2014), the latter trait also observed in NBR and HBR (Simpson et al., 2016). Methane decreased peristaltic velocity and increased ileum contraction amplitude significantly in laboratory animals (Jahng et al., 2012), supporting the observed eosinophil-derived neurotoxin (EDN) increase and micro injuries at the site of small intestine in NBR and HBR variants (Šket et al., 2017a). Due to the slow growing nature of methanogens, slow transit time observed in NBR and HBR would thus assist methanogens in the gut to increase yields from their metabolic activity and adjust their community structure in the long run, resulting in the reported differences between obese and healthy volunteers (Pimentel et al., 2012). The observed relationship between the methane production and the pathogenesis of constipation as well as obesity related syndromes, points to the fact that despite no significant change in Archaeal microbial community was observed in this study, their metabolic activity could be part of manifestations observed in the negatively affected NBR and HBR. The fact that Western-diet constituents exhibited highest methane yields based from the entries in the Methane Yield Database (Murovec et al., 2015) further supported the idea that exercise acted as a safety valve and reduced the availability of substrates for methane production in this study. The effects observed after introduction of the wash-out period support this observation (Debevec et al., 2014; Šket et al., 2017a,b).

Last, fungal diversity and composition was described using 1.7 × 10^4^ fungal hits that were distributed between phylums *Ascomycota* (80.2 ± 5.7%), *Basidiomycota* (15.5 ± 6.5%) and *Microsporidia* (4.3 ± 2.5%) (Figure 2). Similar to the Archaea, there were no significant differences in the fungal taxonomic distribution at various levels of between initial and end-point samples of experimental variants. Fungi of the human microbiome have received least attention so far although many of the species have the potential to conferment various substrates, metabolism of the fungal cell wall in the intestine has the capacity to influence the growth of *E. coli* and other bacteria, while the production of extracellular substances inhibits the growth of pathobionts such as *C. albicans* and/or their yeast-to-hyphae transition (Bojanova and Bordenstein, 2016; Sam et al., 2017). Microbiome and commensal fungi interaction was suggested to be under numerous self-regulating feed-back loops as after the disruption of microbiome the normally commensal elements in the mycobiome can shift their metabolism and turn pathogenic in the long run (Sam et al., 2017), further aggravating the observed negative physiological spiral observed in the PlanHab study (Simpson et al., 2016). The observed significant differences in Archaea and Fungal communities (Mar Rodríguez et al., 2015) in obese and healthy individuals therefore appear to take place at later phases of progressed dysbioses in rather dose dependent manner on the extent of time spent in inactivity.

### Functional annotation of metagenomes

**Figure 3:**
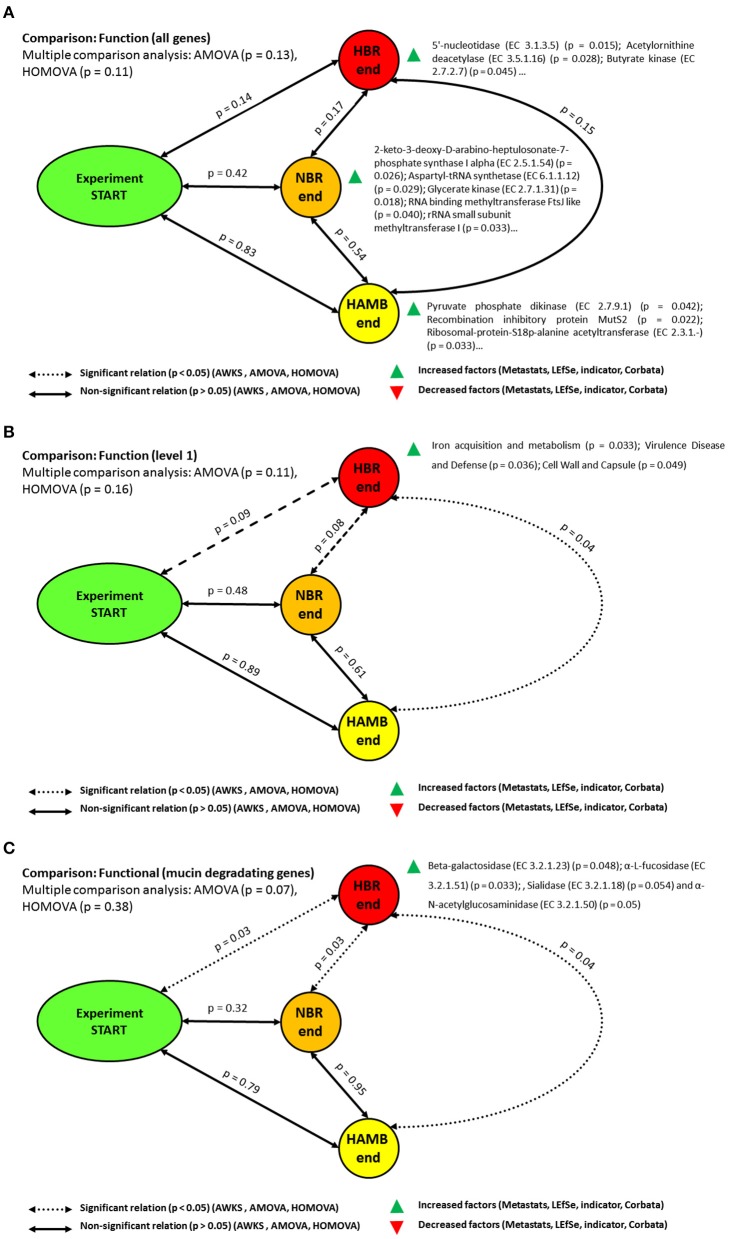
Schematic representation of the significant changes in the functional annotation of microbiomes at the functional level **(A)**, level 1 **(B)** and the category of genes involved in mucin degradation **(C)**. Please consult electronic supplementary material for detailed gene annotation lists.

Altogether, 1.2 × 10^7^ functional hits were assigned to four functional levels. Parsimony and unweighted Unifrac metrics analyses based either on Bray-Curtis, ThetaYC and Jaccard indices did not result in significant differences between initial and end-point experimental variants at the gene level (Table S2). The AMOVA and HOMOVA tests were also not significantly different (*p* = 0.13, *p* = 0.106) between initial and end-point experimental variants after correction for multiple comparisons. In contrast, comparison at the functional level 1 showed that gene groups at the end of HBR variant differed significantly from the end of HAmb variant (*p* = 0.038). Genes coding for proteins involved in iron acquisition and metabolism, cell wall, capsule, virulence, disease and defense were enriched at the end of HBR in comparison to the end of HAmb variant. Although the differences after the correction for multiple comparisons between the start of experiment and the end of HBR variant were on the margin of significance (p = 0.09) and hence deemed not significant, the increase in abundance of gene groups for virulence, disease and defense have been observed (*p* = 0.036; Figure 3, Figure S4). These results suggest that with additional exposure to inactivity (e.g., in ESA 60 day bedrest experiments) significant differences at the level of the genes for cell wall, capsule, virulence, disease and defense could be anticipated. This shows that physical inactivity (lack of activity) *per se* effectively modified the physiological responses of microbial communities toward modified interaction with the host (Johnson et al., 2016; Šket et al., 2017a,b) that eventually resulted in modified microbial community structure and distinct distributions of functional genes. However, no significant differences in richness of microbial functional genes over the course of the PlanHab experiment were detected at any level (*p* > 0.05).

In line with past observations (Šket et al., 2017b) mucin degradation genes differed significantly between endpoints of HAmb and HBR variants (*p* = 0.04), NBR and HBR (*p* = 0.027) variants, and between the start and end of HBR (*p* = 0.032) variants as well. Genes for beta-galactosidase (EC 3.2.1.23) as well for α-L-fucosidase (EC 3.2.1.51), Sialidase (EC 3.2.1.18) and α-N-acetylglucosaminidase (EC 3.2.1.50) were uniformly increased at the end of HBR variant. These enzymes are largely present in the *Bacteroides* genomes, the members of the genus that were significantly enriched at the end of HBR variant (Šket et al., 2017b). The co-identification of significant enrichments for genes coding for capsule proteins and mucin degradation genes are not surprising as these two gene categories are coregulated in *Bacteroides* species (Martens et al., 2009; Šket et al., 2017b) such as *B. thetaiotamicron, B. fragilis* and *B. vulgatus* at the timescale of minutes in response to the environmental characteristics (Johnson et al., 2016). In addition, the environmental polymer distribution (ratios between plant cell wall, microbial exopolysaccharides and host mucin polymers) and temporal (un)availability (constipation, diet shifts) of chemically diverse environmental polymers were shown to affect the preferential use of substrates and hence gene expression (Martens et al., 2009).

Further, ion milieu affects availability, transport and uptake of nutrients of *Bacteroides* and is considered the first sign of innate immune response activity (Martens et al., 2009; Ravcheev et al., 2013). Further, *Bacteroides* are capable of effective switching between the three carbon sources although there exists a preferential use of plant polymers over the host mucus under non-constipated conditions. In addition, the effects of other commensal exopolysaccharides on *Bacteroides thetaiotamicron* gene expression, such as *Bifidobacteria*, were clearly elucidated (Rios-Covian et al., 2013, 2016). *Bacteroides* thus have the capacity to swiftly alter gene expression to match changing substrate availability and environmental fluctuations (Johnson et al., 2016), whereas remarkably few Bacteroidetes were shown to be influenced by host genetics. For the majority of the members of this phylum, environmental factors determine their metabolic activities leading to increased abundance, making them good targets for therapeutic interventions to fine-tune their abundance and metabolic activities.

Genes involved in aerobic and anaerobic respiration have regularly been implicated as those responsible for metabolic endotoxemia of microbial communities in response to increased levels of reactive, oxygen, nitrogen and sulfur species during inflammatory response in obesity related syndromes (Johnson et al., 2016). However, genes for aerobic and anaerobic respiration pathways were not significantly different between the initial and end-point samples in PlanHab experimental variants.

Butyrate synthesis pathways genes at the end of NBR differed significantly from those at the end of HBR (*p* = 0.018). Genes for butyrate kinase were uniformly increased at the end of HBR (*p* = 0.039) whereas butyryl-CoA dehydrogenase gene were increased at the end of NBR. These results are in contrast to the results of no significant difference reported before (Šket et al., 2017a). The main difference stems from the fact that qPCR and amplicon sequencing were used to analyze *buk* and *but* genes, focusing mostly on the most widely reported members belonging to the *Firmicutes* (Šket et al., 2017a). As shot-gun untargeted metagenomics was used in this study, this shows that additional butyrate producing bacteria could be detected and established under the conditions of HBR in addition to those covered by the primers used before (Vital et al., 2013; Šket et al., 2017a). Anyhow, the elevated levels of functional genes were not reflected at the level of increased butyrate concentration in the same samples (Šket et al., 2017a) and ^1^H-NMR metabolomes in this study. The steady state concentrations of C1-C6 short chain fatty acids (SCFA) in feces were also not significantly different between the variants in experiment, thus the exact flux from feces toward the host intestinal cells remains unknown. This is especially intriguing as HBR and NBR participants exhibited significant constipation levels based on Bristol Stool Scale classification (Šket et al., 2017a). A simulation taking into account production and consumption rates from the same diet at different exercise levels (Figure S5) showed the relationship between actual concentration and potential removal rates from fecal matter, congruent with observations of increased SCFA content in obese groups described before.

### Microbial metabolome in the intestinal environment

**Figure 4:**
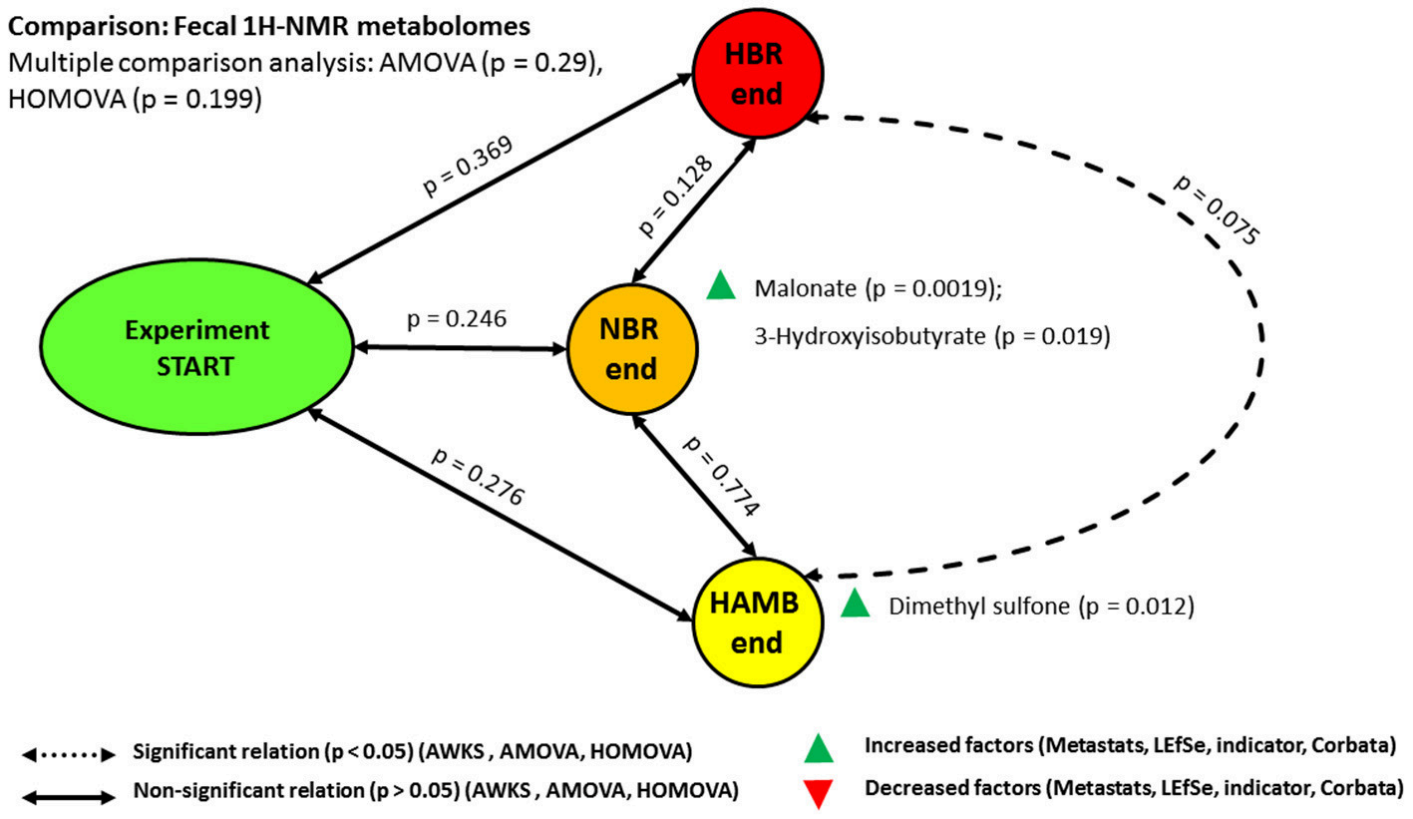
Schematic representation of the relationships between microbial fecal ^1^H-NMR metabolomes. Note the nearly significant relationship between most severely affected HBR and most healthy HAmb.

The analyses of ^1^H-NMR intestinal metabolomes revealed no significant difference between variants over time in response to hypoxia or inactivity (Figure 4). These findings are in-line with our previous measurements of numerous parameters that revealed insignificant changes in the concentration of SCFA, TSOC, sterols and polyphenols, various aromatic compounds through TSOC spectral deconvolution and reducing sugar measurements in the same samples (Šket et al., 2017a,b).

On the other hand, a small set of intestinal parameters (Figure S1) was significantly modified over the course of 21-day experimental phase in NBR, HBR and HAmb variants: Bristol Stool Scale (BSS), EDN, BA, EC, and indole (Šket et al., 2017a,b). As indole is microbial metabolite and the other are host derived this shows that the physiological changes primarily take place at the level of the host and cascade further down to the microbial subsystem that responds by adjusting particular metabolic activities. The predominant lack of change at the level of fecal ^1^H-NMR metabolomes points toward the importance of other levels of interaction between host and microbiome, most probably at the level of proteome, its glycosylation and lipid modification cycles in relation to the negative physiological and psychological symptoms observed in HBR and NBR variants in comparison to HAmb (Debevec et al., 2014; Simpson et al., 2016; Stavrou et al., 2016; Šket et al., 2017a,b; Strewe et al., 2017).

### Electrical conductivity governs intestinal metal availability

**Figure 5:**
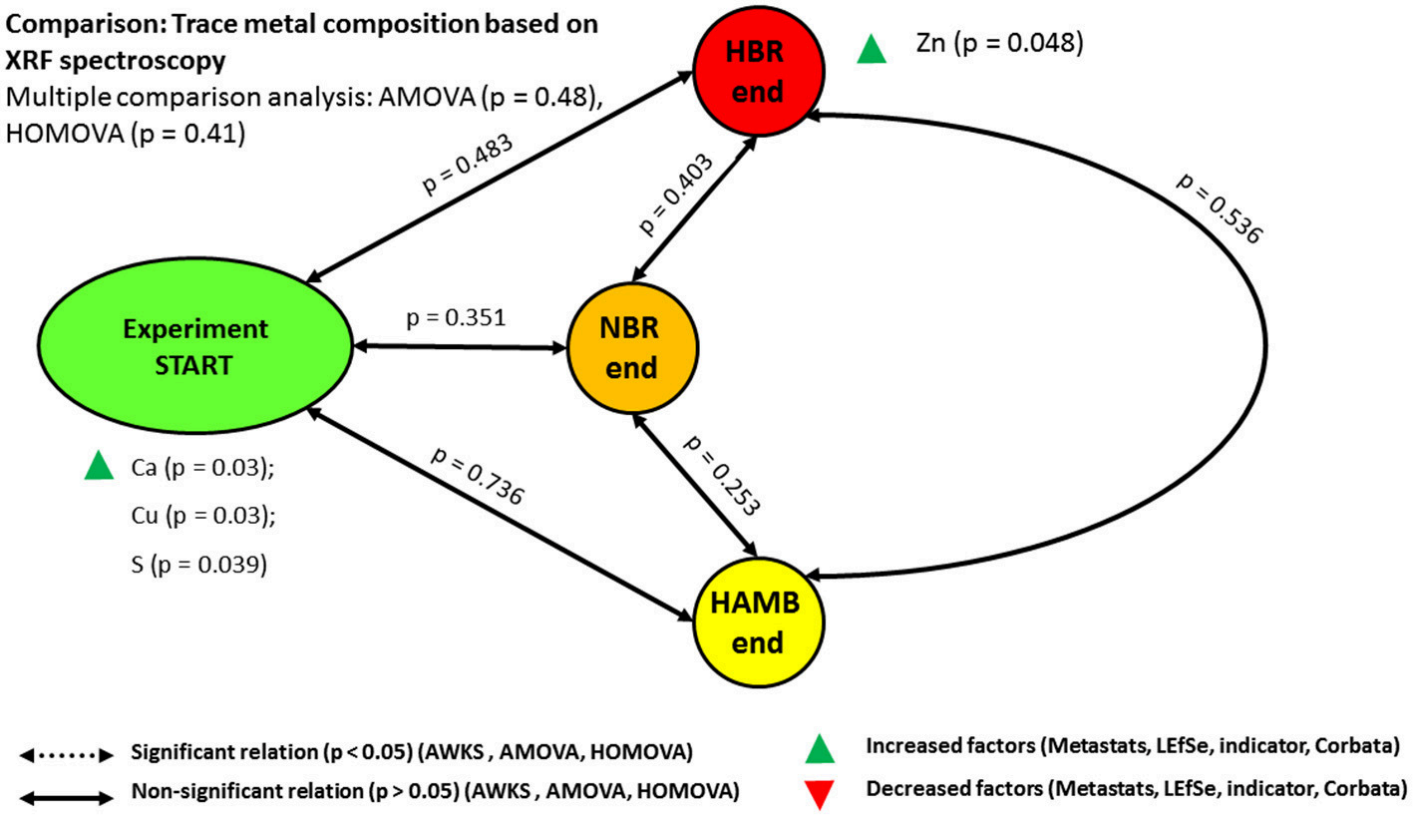
Schematic representation of the relationships between trace metal compositions based on XRF spectroscopy.

XRF measurements of elements and metals in fecal matter revealed no significant difference between variants and time spent in experiment (Figure 5). Elemental and metal content in intestinal tract is of key importance to microbial metabolism, virulence and pathogenesis, their protein synthesis and activity on one hand, and host physiology on the other. On this line of proteomic interaction between the microbiome and the host, microbial siderophores play an important role in sequestering iron and other metals in intestinal tract (Ahmed and Holmström, 2014). In nature, Fe has to compete not only against free protons for the siderophore binding sites but also against other metal ions such as divalent cations, including Cd^2+^, Cu^2+^, Ni^2+^, Pb^2+^, and Zn^2+^ (Albrecht-Gary and Crumbliss, 1998) and trivalent cations, such as Mn^3+^, Co^3+^, and Al^3+^ (Ahmed and Holmström, 2014). There are several studies that have shown that siderophores have an impact on the mobility of these metal ions in the environment (Albrecht-Gary and Crumbliss, 1998; Ahmed and Holmström, 2014; Diaz-Ochoa et al., 2014; Vignesh and Deepe, 2017). Many siderophores are non-ribosomal peptides in addition to the catecholates (phenolates and other aromatic derivatives of lignin degradation and microbial products), hydroxamates and carboxylates (e.g., derivatives of citric acid). Overall binding rate of Fe ions to siderophores decreased by 2–11-fold as electrical conductivity (EC) as a measure of ionic strength increased from ~0.01 to 0.5 M (Ahmed and Holmström, 2014). The increased EC in NBR and HBR as part of innate immune system indicated the attempts of the host to systematically decrease the availability of metals to microbes. Over the course of the PlanHab experiment NBR and HBR participants exhibited elevated EC (Šket et al., 2017b) at comparable metal contents in their diet as observed in this study, coinciding with the increased inflammatory responses (Simpson et al., 2016).

In addition to promoting microbial growth by binding metals, there is emerging evidence of clinical relevance that microbial siderophores can effectively modulate the host response. It was previously shown that siderophores secreted by enteric pathogens have the capacity to cause hypoxia-dependent activation of HIF-1 in the Peyer's patches and in human epithelial and endothelial cells, a transcription factor that plays pivotal roles during infection (Behnsen and Raffatellu, 2016; Šket et al., 2017a,b).

### Bayesian network as a model of interactions at systems level

**Figure 6:**
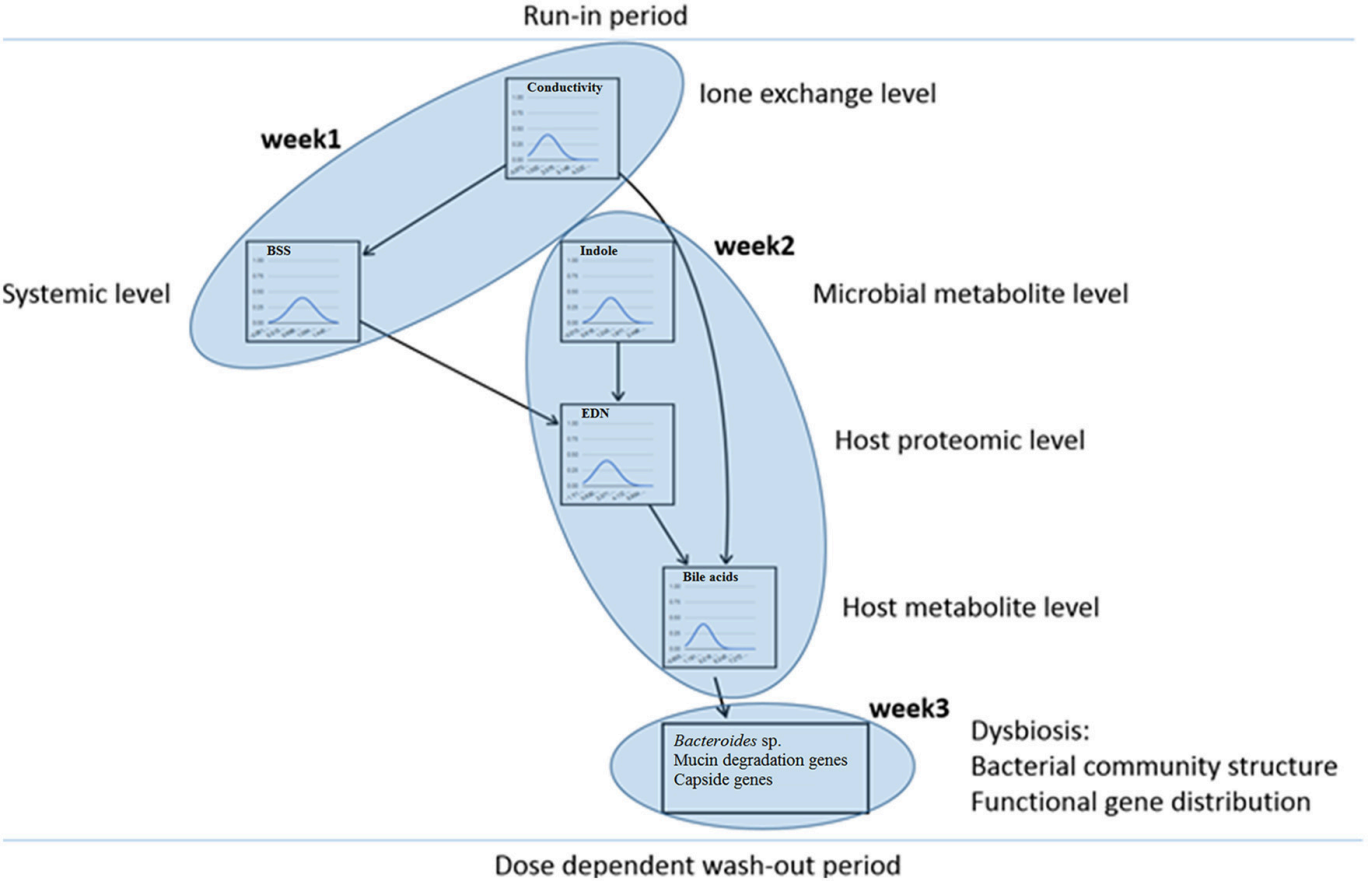
Bayesian network analysis. The intestinal parameters that differed significantly over the course of the PlanHab experiment were used to derive a model of human physiology responses establishing hierarchy in the measured parameters.

A number of parameters (*n* = 261) was monitored over the course of the PlanHab experiment and a database containing these data was interrogated (Šket et al., 2017a,b). Only the intestinal parameters that differed significantly over the course of the PlanHab experiment were used to derive a model of human physiology responses (Figure 6; Figure S1) establishing hierarchy in the measured parameters for the first time. Exploration of the network showed that changes in the parameter levels resulted in reproduction of the responses observed within the PlanHab study (Debevec et al., 2014; Simpson et al., 2016). For instance, decrease in fecal electrical conductivity resulted in increased BSS (no constipation) and reduced bile acids (BA) levels. Secondly, increased indole concentrations at retained lower electrical conductivity resulted in lowered intestinal inflammation (EDN) levels. On the other hand, if BA levels were increased within the network, EDN and conductivity were increased as well, whereas BSS levels declined further toward constipation and reduced indole production. However, this link between microbial indole production and inflammation marker EDN suggested that a non-linear responses take place over the network as small changes in indole levels exhibited unexpectedly large effects on BA content.

In addition, the tiers within the Bayes network corresponded to the weeks within which significant parameters appeared within the experiment (Figure 6) and providing for the first time a model describing the initial steps within the human microbiome and its host system in response to the acute inactivity: (i) decision to decrease the level of physical activity gave rise to a cascade-like modifications in human physiology over time; (ii) changes in host physiology preceded changes in human microbiome structure; (iii) changes in host physiology and intestinal parameters guided human microbiome physiology and activity; (iv) prolonged changes in host physiology supported distinct microbiome activity and resulted in modified microbiome structure next to aggravated physiological status of the host; and (v), the introduction of a lifestyle change in the form of the exercise during the wash-out period at the end of 21-day experiment effectively resulted in alleviation of the developed negative symptoms in human physiology, also in dose dependent manner (Debevec et al., 2014; Simpson et al., 2016).

## Discussion

### Exercise trains microbiome functionality

Elevated levels of indole and its derivatives, tryptophan-derived microbial metabolites, observed in HAmb, were shown to promote host intestinal tract barrier integrity and expression of anti-inflammatory interleukins (e.g., IL-10), inhibiting proinflammatory TNF-α and NF-κB signaling (Steinmeyer et al., 2015). Consequently, translocation of antigens and pathogens was most probably decreased, including reduced LPS infiltration into blood stream, resulting in reduced endotoxemia and host inflammation (Rooks and Garrett, 2016).

The importance of exercise is linked to many-faceted nature of human exercise: the vertical hydrostatic gradients within body (Debevec et al., 2014), electrolyte losses from sweating, posture energy consumption (Miles-Chan and Dulloo, 2017), significant changes in the host muscle and circulating metabolome, that all take place at the time scale of minutes following resistance exercise (Berton et al., 2016). All these are most probably associated with positive effects of the energy-consumption-through-exercise lifestyle in HAmb and are reflected as healthy levels of intestinal EC, BA, lack of constipation, lack of intestinal inflammation (EDN) and increased indole levels (Šket et al., 2017a,b).

From this perspective, it has become increasingly evident that the gut microbiome is an essential supplier of extensive range of metabolites that influence mitochondrial function and biogenesis within the skeletal muscle to stabilize host metabolism and its corresponding production of various metabolites, proteins and signaling molecules (Franco-Obregón and Gilbert, 2017), while contractions of skeletal muscles increase fatty acid oxidation and promote mitochondrial biogenesis as well as microbiome structural and functional adaptations (Clarke et al., 2014; Clark and Mach, 2017; Šket et al., 2017b). Hence, a growing body of evidence supports the idea that exercise provides the systemic environment of signal products and metabolites originating from microorganisms as well (Garagnani et al., 2014) that modulate intestinal barrier function, mucosal immune response and mitochondrial reactive oxygen species production, next to acute inflammatory responses (Clark and Mach, 2017). These parameters functionally inhibit, thermodynamically confine or make microbiota adapt to metabolic demands of physically active lifestyle of the host, carrying out a set of physiological functions (Estaki et al., 2016). From the extensively distinct microbial assemblies and rather undirected changes in their structure it follows that the metabolic function of microbiome is more important to the host than the identity of the species providing it (Marchesi et al., 2015; Beaumont et al., 2017). This was reflected in the finding that the diversity at various taxonomic and functional levels was not significantly affected within the 4-week time scale of the PlanHab experiment.

### The split personality of *Bacteroides*: friend and foe

As members of the phylum Bacteriodetes are highly adapted to life in rapidly changing environment (Johnson et al., 2016), it is conceivable, that their exposure to different environmental conditions in HBR (host inactivity and hypoxia) modified microbial physiology and provided sufficient competitive advantage that resulted in their relative increase in microbial community. However, inferring metabolic roles by taxonomic classification alone by making the association of specific functional roles to entire taxonomic groups is difficult, because phylogenetically closely related organisms were shown to contain differentially organized genomes and hence exhibit very different metabolism (Bauer et al., 2015; Johnson et al., 2016), and hence could produce rather distinct metabolic fingerprints (Beaumont et al., 2017). Although the host inactivity resulted in significant modification of some of the environmental parameters of intestinal tract, this has apparently primarily directed microbial physiology toward degradation of host mucin polymers and concomitant upregulation of inflammagenic capsid proteins at the same time (Martens et al., 2009). The increased abundance of mucin degradation and capside related genes in HBR metagenomes (this study) supports this observation. The increased BA and EC levels in feces point toward host attempts to counteract microbial activities, overgrowth and production of metabolites.

Although *Bacteroides* are tolerant to BA, their increased abundance in HBR was associated with the abnormal host glucose metabolism and insulin resistance before (Qin et al., 2012; Karlsson et al., 2013; Zeevi et al., 2015). In parallel, the same positive relationship between insulin resistance and BA was observed in HBR, whereas this relationship was present in NBR devoid of significant community change.

As the increase in EC decreases availability of iron in intestinal system by default, this forces *Bacteroides* to activate hemolysin production in order to perforate host cells to provide haem inflow (Robertson et al., 2006). Second, capside genes code for proteins that bind major clotting proteins from human plasma and prolong the intrinsic coagulation time or prevent clot formation (Murphy et al., 2011), whereas accompanying polysaccharide A activates immune system leading to increased blood inflow (Elhenawy et al., 2014). Capside and mucin degradation genes are coregulated, and the latter have the potential to decrease mucin thickness, crosslinking, structure, porosity, influencing the diffusion gradients and immune contacts (Martens et al., 2009), especially under constipated conditions, observed in HBR and NBR. Third, glycosidases, proteases, polysaccharide A and polysaccharide B, outer membrane proteins, phospholipids, sphingolipids, exotoxins are preferentially packed into outer membrane vesicles (OMV) released by many *Bacteroides*, targeting complex polysaccharides from diet or human origin, such as mucus glycans, allowing *Bacteroides* to modulate host pathways (Elhenawy et al., 2014). Lastly, inactivity and hypoxia lead to suboptimal mitochondrial function and release of reactive oxygen species that effectively damage microbial cells, releasing LPS (lipid A). These are taken up by host cells under high BA and EC conditions through leaky tight junctions, microbial perforations, intestinal abrasions (Šket et al., 2017a,b), chylomicrons responsible for dietary lipid uptake, or even OMV (Elhenawy et al., 2014), resulting in the initialization of inflammation-based processes linked with insulin resistance (Boulangé et al., 2016; Rowland et al., 2017), a condition observed in HBR and NBR participants. Consequently, the expression of inflammatory molecules was triggered by increased systemic levels of endotoxins, including nuclear factor κB (NF-κB) (Simpson et al., 2016; Šket et al., 2017a,b).

In humans, the circulating endotoxin levels in blood were shown to increase by 20% in individuals with increased glucose intolerance in comparison to healthy individuals (Cani et al., 2007; Li et al., 2011; Boulangé et al., 2016). This is in line with the observation that increased BA and EC in HBR and NBR variants gave rise to increased intestinal translocation into plasma, causing the observed negative inflammatory dose dependent physiological symptoms in HBR and NBR (Debevec et al., 2014; Simpson et al., 2016).

### Derangements after inactivity

Metabolic (BA), proteomic (EDN), ionic (EC), systemic (BSS) adaptations observed before (Šket et al., 2017a,b) take place in parallel with physiological symptoms (Debevec et al., 2016; Simpson et al., 2016; Strewe et al., 2017) but precede the dysbiosis with the time window of 2–3 weeks. We hypothesize that this microbiome persistence has evolved to act as a “buffer” contributing to the stability of metabolic homeostasis over prolonged periods of time, by preventing overly fluctuating metabolic responses to incidental nutritional or environmental signals (Thaiss et al., 2016; Beaumont et al., 2017). Evolutionary adaptations, such as starvation during winter hibernation bouts, have the capacity to induce seasonal cyclic changes in microbial community structure, insulin resistance, hyperglycemia, and increase in *Bacteroides* content. In addition, these events predispose the host toward effective weight-gain during summer feast period, a trait apparently broadly conserved in evolutionary diverse range of mammalian species (Carey and Martin, 2003; Carey, 2013; Sommer et al., 2016) as recently proposed (Šket et al., 2017b). In addition, the onset of wash-out period (HBR and NBR variants, i.e., 14 and 10 days, respectively; Debevec et al., 2014; Šket et al., 2017a) is rather symmetric to the end of hibernation bout (Carey and Martin, 2003; Carey, 2013; Sommer et al., 2016), as largely the same negative symptoms were alleviated after reintroduction of exercise irrespective of mammalian species. These data suggest that the yo-yo dieting and the effects on weight gain (Thaiss et al., 2016) are not a human peculiarity but rather a common evolutionary adaptation of mammals to survive cyclic food shortage.

## Conclusions

The observed progressive decrease in defecation frequency and concomitant increase in the electrical conductivity (EC) preceded or took place in absence of significant taxonomic and functional gene rearrangements in bacterial, archaeal and fungal microbial communities, their metabolomes and intestinal metal profiles.

The fact that the genus *Bacteroides* and proteins involved in iron acquisition and metabolism, cell wall, capsule, virulence and mucin degradation were enriched elucidates for the first time the initial mechanism of step-by-step development of the observed negative physiological symptoms of the host. These changes were largely induced by onset of inactivity (NBR), compounded by hypoxia (HBR) and relieved by exercise despite hypoxia (HAmb). The onset of the wash-out period in the PlanHab experiment corresponded to a profound life-style change that resulted in immediate amelioration of the negative physiological symptoms, indicating that exercise acted as an important parameter apparently downplaying microbial physiology and metabolic activities involved in degradation of the intestinal mucus layer, iron scavenging and proinflammatory immune crosstalk.

## Ethics statement

This study was carried out in accordance with the recommendations of European Space Agency's standardization plan for bed rest studies and National Committee for Medical Ethics at the Ministry of Health of the Republic of Slovenia with written informed consent from all subjects. All subjects gave written informed consent in accordance with the Declaration of Helsinki. The protocol was approved by the National Committee for Medical Ethics at the Ministry of Health of the Republic of Slovenia.

## Data availability

All obtained reads were deposited on MG-RAST database server under project accession number mgp17406 (http://metagenomics.anl.gov/linkin.cgi?project=mgp17406). All metabolomic and trace metal profile data are available from the corresponding author upon request.

## Author contributions

BS provided the concept for microbiome and metabolome analysis. TD, BS collected samples. BS, MS, RŠ, AS designed microbiome analyses. RŠ, BS, SK, ZP, BM, KV, DM, KP, IM, OE conducted research. RŠ, BS analyzed the data. RŠ, BS, BM provided necessary code. RŠ, BS provided statistical analyses. BS drafted manuscript. All authors provided intellectual content at various stages of project development and manuscript preparation.

### Conflict of interest statement

The authors declare that the research was conducted in the absence of any commercial or financial relationships that could be construed as a potential conflict of interest. The reviewer AL and handling Editor declared their shared affiliation.

## Abbreviations

^1^H-NMR: Proton nuclear magnetic resonance
BA: Bile acids
BSS: Bristol stool scale
EC: Electrical conductivity
EDN: Eosinophil-derived neurotoxin
ESA: European Space Agency
HAmb: Normobaric hypoxic ambulatory confinement
HBR: Normobaric hypoxic bed rest
HSQC: Heteronuclear single quantum coherence spectrum
NBR: Normobaric normoxic bed rest
SCFA: C1-C6 short chain fatty acids
TOCSY: Total correlated spectrum
TSOC: Total dissolved organic matter concentration
XRF: X-ray fluorescence spectrometry.

## References

[B1] Ahmed E. Holmström S. J. M. (2014). Siderophores in environmental research: roles and applications. Microb. Biotechnol. 7, 196–208. 10.1111/1751-7915.12117 24576157 PMC3992016

[B2] Albrecht-Gary A. M. Crumbliss A. L. (1998). Coordination chemistry of siderophores: thermodynamics and kinetics of iron chelation and release. Met. Ions Biol. Syst. 35, 239–327. 9444763

[B3] Barton W. Penney N. C. Cronin O. Garcia-Perez I. Molloy M. G. Holmes E.. (2017). The microbiome of professional athletes differs from that of more sedentary subjects in composition and particularly at the functional metabolic level. Gut. [Epub ahead of print]. 10.1136/gutjnl-2016-313627 28360096

[B4] Bauer E. Laczny C. C. Magnusdottir S. Wilmes P. Thiele I. (2015). Phenotypic differentiation of gastrointestinal microbes is reflected in their encoded metabolic repertoires. Microbiome 3:55. 10.1186/s40168-015-0121-6 26617277 PMC4663747

[B5] Beaumont M. Portune K. J. Steuer N. Lan A. Cerrudo V. Audebert M.. (2017). Quantity and source of dietary protein influence metabolite production by gut microbiota and rectal mucosa gene expression: a randomized, parallel, double-blind trial in overweight humans. Am. J. Clin. Nutr. 106, 1005–1019. 10.3945/ajcn.117.158816 28903954

[B6] Beckonert O. P. Keun H. C. Ebbels T. M. D. Bundy J. G. Holmes E. Lindon J. C.. (2007). Metabolic profiling, metabolomic and metabonomic procedures for NMR spectroscopy of urine, plasma, serum and tissue extracts. Nat. Protoc. 2, 2692–2703. 10.1038/nprot.2007.376 18007604

[B7] Behnsen J. Raffatellu M. (2016). Siderophores: more than stealing iron. MBio 7:e01906-16. 10.1128/mBio.01906-16 27935843 PMC5111413

[B8] Benjamini Y. Hochberg Y. (1995). Controlling the false discovery rate: a practical and powerful approach to multiple testing. J. R. Stat. Soc. Ser. B 57, 289–300.

[B9] Benjamini Y. Yekutieli D. (2001). The control of the false discovery rate in multiple testing under depencency. Ann. Stat. 29, 1165–1188. 10.1214/aos/1013699998

[B10] Berton R. Conceição M. S. Libardi C. A. Canevarolo R. R. Gáspari A. F. Chacon-Mikahil M. P. T.. (2016). Metabolic time-course response after resistance exercise: a metabolomics approach. J. Sports Sci. 35, 1211–1218. 10.1080/02640414.2016.1218035 27686013

[B11] Bojanova D. P. Bordenstein S. R. (2016). Fecal transplants: what is being transferred? PLoS Biol. 14:e1002503. 10.1371/journal.pbio.1002503 27404502 PMC4942072

[B12] Boulangé C. L. Neves A. L. Chilloux J. Nicholson J. K. Dumas M.-E. (2016). Impact of the gut microbiota on inflammation, obesity, and metabolic disease. Genome Med. 8:42. 10.1186/s13073-016-0303-2 27098727 PMC4839080

[B13] Candela M. Biagi E. Maccaferri S. Turroni S. Brigidi P. (2012). Intestinal microbiota is a plastic factor responding to environmental changes. Trends Microbiol. 20, 385–391. 10.1016/j.tim.2012.05.003 22672911

[B14] Cani P. D. Amar J. Iglesias M. A. Poggi M. Knauf C. Bastelica D.. (2007). Metabolic endotoxemia initiates obesity and insulin resistance. Diabetes 56, 1761–1772. 10.2337/db06-1491 17456850

[B15] Carey H. V. Andrews M. T. Martin S. L. (2003). Mammalian hibernation: cellular and molecular responses to depressed metabolism and low temperature. Physiol. Rev. 83, 1153–1181. 10.1152/physrev.00008.2003 14506303

[B16] Carey H. V. Walters W. A. and Knight, R. (2013). Seasonal restructuring of the ground squirrel gut microbiota over the annual hibernation cycle. Am. J. Physiol. Regul. Integr. Comp. Physiol. 304, R33–R42. 10.1152/ajpregu.00387.2012 23152108 PMC3543654

[B17] Clark A. Mach N. (2017). The crosstalk between the gut microbiota and mitochondria during exercise. Front. Physiol. 8:319. 10.3389/fphys.2017.00319 28579962 PMC5437217

[B18] Clarke S. F. Murphy E. F. O'Sullivan O. Lucey A. J. Humphreys M. Hogan A.. (2014). Exercise and associated dietary extremes impact on gut microbial diversity. Gut 63, 1913–1920. 10.1136/gutjnl-2013-306541 25021423

[B19] Debevec T. Bali T. C. Simpson E. J. Macdonald I. A. Eiken O. Mekjavic I. B. (2014). Separate and combined effects of 21-day bed rest and hypoxic confinement on body composition. Eur. J. Appl. Physiol. 114, 2411–2425. 10.1007/s00421-014-2963-1 25091855

[B20] Debevec T. Simpson E. J. Mekjavic I. B. Eiken O. Macdonald I. A. (2016). Effects of prolonged hypoxia and bed rest on appetite and appetite-related hormones. Appetite 107, 28–37. 10.1016/j.appet.2016.07.005 27395413

[B21] Diaz-Ochoa V. E. Jellbauer S. Klaus S. Raffatellu M. (2014). Transition metal ions at the crossroads of mucosal immunity and microbial pathogenesis. Front. Cell. Infect. Microbiol. 4:2. 10.3389/fcimb.2014.00002 24478990 PMC3900919

[B22] Elhenawy W. Debelyy M. O. Feldman M. F. (2014). Preferential packing of acidic glycosidases and proteases into Bacteroides outer membrane vesicles. MBio 5:e00909-14. 10.1128/mBio.00909-14 24618254 PMC3952158

[B23] ESA (2009). Standardization of Bed Rest Study Conditions (Version 1.5). (ESTEC contract number 20187/06/NL/VJ). Paris: European Space Agency.

[B24] Estaki M. Pither J. Baumeister P. Little J. P. Gill S. K. Ghosh S.. (2016). Cardiorespiratory fitness as a predictor of intestinal microbial diversity and distinct metagenomic functions. Microbiome 4, 42. 10.1186/s40168-016-0189-7 27502158 PMC4976518

[B25] Franco-Obregón A. Gilbert J. (2017). The microbiome-mitochondrion connection: common ancestries, common mechanisms, common goals. mSystems 2:e00018-17. 10.1128/mSystems.00018-17 28497122 PMC5425687

[B26] Garagnani P. Pirazzini C. Giuliani C. Candela M. Brigidi P. Sevini F.. (2014). The three genetics (nuclear DNA, mitochondrial DNA, and gut microbiome) of longevity in humans considered as metaorganisms. BioMed Res. Int. BioMed Res. Int. 2014:e560340. 10.1155/2014/560340 24868529 PMC4017728

[B27] Gregor M. F. Hotamisligil G. S. (2011). Inflammatory mechanisms in obesity. Annu. Rev. Immunol. 29, 415–445. 10.1146/annurev-immunol-031210-101322 21219177

[B28] Jahng J. Jung I. S. Choi E. J. Conklin J. L. Park H. (2012). The effects of methane and hydrogen gases produced by enteric bacteria on ileal motility and colonic transit time. Neurogastroenterol. Motil. 24, 185–90, e92. 10.1111/j.1365-2982.2011.01819.x 22097886

[B29] Johnson E. L. Heaver S. L. Walters W. A. Ley R. E. (2016). Microbiome and metabolic disease: revisiting the bacterial phylum Bacteroidetes. J. Mol. Med. 95, 1–8. 10.1007/s00109-016-1492-2 27900395 PMC5187364

[B30] Kamada N. Chen G. Y. Inohara N. Núñez G. (2013). Control of pathogens and pathobionts by the gut microbiota. Nat. Immunol. 14, 685–690. 10.1038/ni.2608 23778796 PMC4083503

[B31] Karlsson F. Tremaroli V. Nookaew I. Bergström G. Behre C. Fagerberg B.. (2013). Gut metagenome in European women with normal, impaired and diabetic glucose control. Nature 498, 99–103. 10.1038/nature12198 23719380

[B32] Kolbl S. Forte-Tavčer P. Stres B. (2017). Potential for valorization of dehydrated paper pulp sludge for biogas production: addition of selected hydrolytic enzymes in semi-continuous anaerobic digestion assays. Energy 126, 326–334. 10.1016/j.energy.2017.03.050

[B33] Kump P. Nečemer M. Smodiš B. Jačimović R. (1996). Multielement analysis of rubber samples by X-ray fluorescence. Appl. Spectrosc. 50, 1373–1377. 10.1366/0003702963904674

[B34] Legendre P. Legendre L. F. J. (2012). Numerical Ecology, 3rd Edn. Amsterdam: Elsevier.

[B35] Li K. Bihan M. Methé B. A. (2013). Analyses of the stability and core taxonomic memberships of the human microbiome. PLoS ONE 8:e63139. 10.1371/journal.pone.0063139 23671663 PMC3646044

[B36] Li Y. Innocentin S. Withers D. R. Roberts N. A. Gallagher A. R. Grigorieva E. F.. (2011). Exogenous stimuli maintain intraepithelial lymphocytes via aryl hydrocarbon receptor activation. Cell 147, 629–640. 10.1016/j.cell.2011.09.025 21999944

[B37] Likar M. Vogel-Mikuš K. Potisek M. Hančević K. Radić T. Nečemer M.. (2015). Importance of soil and vineyard management in the determination of grapevine mineral composition. Sci. Total Environ. 505, 724–731. 10.1016/j.scitotenv.2014.10.057 25461075

[B38] Lumeng C. N. Saltiel A. R. (2011). Review series Inflammatory links between obesity and metabolic disease. J. Clin. Invest. 121, 2111–2117. 10.1172/JCI57132 21633179 PMC3104776

[B39] Marchesi J. R. Adams D. H. Fava F. Hermes G. D. A. Hirschfield G. M. Hold G.. (2015). The gut microbiota and host health: a new clinical frontier. Gut 65, 330–339. 10.1136/gutjnl-2015-309990 26338727 PMC4752653

[B40] Mar Rodríguez M. Pérez D. Javier Chaves F. Esteve E. Marin-Garcia P. Xifra G.. (2015). Obesity changes the human gut mycobiome. Sci. Rep. 5:14600. 10.1038/srep14600 26455903 PMC4600977

[B41] Martens E. C. Roth R. Heuser J. E. Gordon J. I. (2009). Coordinate regulation of glycan degradation and polysaccharide capsule biosynthesis by a prominent human gut symbiont. J. Biol. Chem. 284, 18445–18457. 10.1074/jbc.M109.008094 19403529 PMC2709373

[B42] Mathur R. Goyal D. Kim G. Barlow G. M. Chua K. S. Pimentel M. (2014). Methane-producing human subjects have higher serum glucose levels during oral glucose challenge than non-methane producers: a pilot study of the effects of enteric methanogens on glycemic regulation. Res. J. Endocrinol. Metab. 2:2 10.7243/2053-3640-2-2

[B43] Meyer F. Paarmann D. D'Souza M. Olson R. Glass E. M. Kubal M.. (2008). The metagenomics RAST server - a public resource for the automatic phylogenetic and functional analysis of metagenomes. BMC Bioinformatics 9:386. 10.1186/1471-2105-9-386. 18803844 PMC2563014

[B44] Miles-Chan J. L. Dulloo A. G. (2017). Posture allocation revisited: breaking the sedentary threshold of energy expenditure for obesity management. Front. Physiol. 8:420. 10.3389/fphys.2017.00420 28690547 PMC5479887

[B45] Monda V. Villano I. Messina A. Valenzano A. Esposito T. Moscatelli F.. (2017). Exercise modifies the gut microbiota with positive health effects. Oxid. Med. Cell. Longev. 2017:3831972. 10.1155/2017/3831972 28357027 PMC5357536

[B46] Murovec B. Kolbl S. Stres B. (2015). Methane yield database: online infrastructure and bioresource for methane yield data and related metadata. Bioresour. Technol. 189, 217–223. 10.1016/j.biortech.2015.04.021 25898082

[B47] Murphy E. C. Mörgelin M. Cooney J. C. Frick I. M. (2011). Interaction of bacteroides fragilis and bacteroides thetaiotaomicron with the kallikrein-kinin system. Microbiology 157, 2094–2105. 10.1099/mic.0.046862-0 21527472 PMC3167891

[B48] Nečemer M. Kump P. Ščančar J. Jaćimović R. Simčič J. Pelicon P. (2008). Application of X-ray fluorescence analytical techniques in phytoremediation and plant biology studies. Spectrochim. Acta B At. Spectrosc. 63, 1240–1247. 10.1016/j.sab.2008.07.006

[B49] Perry R. Samuel V. Petersen K. Shulman G. (2014). The role of hepatic lipids in hepatic insulin resistance and type 2 diabetes. Nature 510, 84–91. 10.1038/nature13478 24899308 PMC4489847

[B50] Pimentel M. Gunsalus R. P. Rao S. S. Zhang H. (2012). Methanogens in human health and disease. Am. J. Gastroenterol. Suppl. 1, 28–33. 10.1038/ajgsup.2012.6

[B51] Qin J. Li Y. Cai Z. Li S. Zhu J. Zhang F.. (2012). A metagenome-wide association study of gut microbiota in type 2 diabetes. Nature 490, 55–60. 10.1038/nature11450 23023125

[B52] Ravcheev D. A. Godzik A. Osterman A. L. Rodionov D. A. (2013). Polysaccharides utilization in human gut bacterium Bacteroides thetaiotaomicron: comparative genomics reconstruction of metabolic and regulatory networks. BMC Genomics 14:873. 10.1186/1471-2164-14-873 24330590 PMC3878776

[B53] Rios-Covian D. Arboleya S. Hernandez-Barranco A. M. Alvarez-Buylla J. R. Ruas-Madiedo P. Gueimonde M.. (2013). Interactions between Bifidobacterium and Bacteroides species in cofermentations are affected by carbon sources, including exopolysaccharides produced by bifidobacteria. Appl. Environ. Microbiol. 79, 7518–7524. 10.1128/AEM.02545-13 24077708 PMC3837738

[B54] Rios-Covian D. Cuesta I. Alvarez-Buylla J. R. Ruas-Madiedo P. Gueimonde M. de Los Reyes-Gavilán C. G. (2016). Bacteroides fragilis metabolises exopolysaccharides produced by bifidobacteria. BMC Microbiol. 16:150. 10.1186/s12866-016-0773-9 27418149 PMC4946188

[B55] Rittweger J. Debevec T. Frings-Meuthen P. Lau P. Mittag U. Ganse B.. (2016). On the combined effects of normobaric hypoxia and bed rest upon bone and mineral metabolism: results from the PlanHab study. Bone 91, 130–138. 10.1016/j.bone.2016.07.013 27443510

[B56] Robertson K. P. Smith C. J. Gough A. M. Rocha E. R. (2006). Characterization of Bacteroides fragilis hemolysins and regulation and synergistic interactions of HlyA and HlyB. Infect. Immun. 74, 2304–2316. 10.1128/IAI.74.4.2304-2316.2006 16552061 PMC1418898

[B57] Rooks M. G. Garrett W. S. (2016). Gut microbiota, metabolites and host immunity. Nat. Rev. Immunol. 16, 341–352. 10.1038/nri.2016.42 27231050 PMC5541232

[B58] Rowland I. Gibson G. Heinken A. Scott K. Swann J. Thiele I.. (2017). Gut microbiota functions: metabolism of nutrients and other food components. Eur. J. Nutr. 57, 1–24. 10.1007/s00394-017-1445-8 28393285 PMC5847071

[B59] Saltiel A. R. Kahn C. R. (2001). Insulin signalling and the regulation of glucose and lipid metabolism. Nature 414, 799–806. 10.1038/414799a 11742412

[B60] Sam Q. H. Chang M. W. Chai L. Y. A. (2017). The fungal mycobiome and its interaction with gut bacteria in the host. Int. J. Mol. Sci. 18:E330. 10.3390/ijms18020330 28165395 PMC5343866

[B61] Schloss P. D. Westcott S. L. Ryabin T. Hall J. R. Hartmann M. Hollister E. B.. (2009). Introducing mothur: open-source, platform-independent, community-supported software for describing and comparing microbial communities. Appl. Environ. Microbiol. 75, 7537–7541. 10.1128/AEM.01541-09 19801464 PMC2786419

[B62] Schneider H. Blaut M. (2000). Anaerobic degradation of flavonoids by Eubacterium ramulus. Arch. Microbiol. 173, 71–75. 10.1007/s002030050010 10648107

[B63] Sell H. Habich C. Eckel J. (2012). Adaptive immunity in obesity and insulin resistance. Nat. Rev. Endocrinol. 8, 709–716. 10.1038/nrendo.2012.114 22847239

[B64] Simpson E. J. Debevec T. Eiken O. Mekjavic I. B. Macdonald I. A. (2016). The combined and separate effects of 16 days bed rest and normobaric hypoxic confinement on circulating lipids and indices of insulin sensitivity in healthy men. J. Appl. Physiol. 120, 947–955. 10.1152/japplphysiol.00897.2015 26769956 PMC4835909

[B65] Šket R. Treichel N. Debevec T. Mekjavic I. B. Eiken O. Schloter M.. (2017a). Hypoxia and inactivity related physiological changes (constipation, inflammation) are not reflected at the level of gut metabolites and butyrate producing microbial community: the PlanHab study. Front Physiol 8:250. 10.3389/fphys.2017.00250 28522975 PMC5416748

[B66] Šket R. Treichel N. Kublik S. Debevec T. Eiken O. Mekjavic I. B.. (2017b). Hypoxia and inactivity related physiological changes precede or take place in absence of significant rearrangements in bacterial community structure: the PlanHab randomized pilot trial study. PLoS ONE 12:e0188556. 10.1371/journal.pone.0188556 29211803 PMC5718606

[B67] Sommer F. Ståhlman M. Ilkayeva O. Arnemo J. M. Kindberg J. Josefsson J.. (2016). The gut microbiota modulates energy metabolism in the hibernating brown bear Ursus arctos. Cell Rep. 14, 1655–1661. 10.1016/j.celrep.2016.01.026 26854221

[B68] Stavrou N. A. Debevec T. Eiken O. Mekjavič I. B. (2016). Hypoxia worsens affective responses and feeling of fatigue during prolonged inactivity, in Joint Life Science Meeting “Life in Space for Life on Earth” (Paper Presentet at 14th European Life Sciences Symposium and 37th Annual International Gravitational Physiology Meeting) (Toulouse), 187.

[B69] Steinmeyer S. Lee K. Jayaraman A. Alaniz R. C. (2015). Microbiota metabolite regulation of host immune homeostasis: a mechanistic missing link. Curr. Allergy Asthma Rep. 15:24. 10.1007/s11882-015-0524-2 26139332

[B70] Strewe C. Zeller R. Feuerecker M. Hoerl M. Kumprej I. Crispin A. (2017). PlanHab study: assessment of psycho-neuroendocrine function in male subjects during 21 days of normobaric hypoxia and bed rest. Stress 20, 1–21. 10.1080/10253890.2017.1292246 28166699

[B71] Thaiss C. A. Itav S. Rothschild D. Meijer M. Levy M. Moresi C. (2016). Persistent microbiome alterations modulate the rate of post-dieting weight regain. Nature 540, 544–551. 10.1038/nature20796 27906159

[B72] Triantafyllou K. Chang C. Pimentel M. (2014). Methanogens, methane and gastrointestinal motility. J. Neurogastroenterol. Motil. 20, 31–40. 10.5056/jnm.2014.20.1.31 24466443 PMC3895606

[B73] Vignesh K. S. Deepe G. S. (2017). Metallothioneins: emerging modulators in immunity and infection. Int. J. Mol. Sci. 18:E2197 10.3390/ijms18102197 29065550 PMC5666878

[B74] Vital M. Penton C. R. Wang Q. Young V. B. Antonopoulos D. A. Sogin M. L.. (2013). A gene-targeted approach to investigate the intestinal butyrate-producing bacterial community. Microbiome 1:8. 10.1186/2049-2618-1-8 24451334 PMC4126176

[B75] Wishart D. S. Jewison T. Guo A. C. Wilson M. Knox C. Liu Y.. (2013). HMDB 3.0-The human metabolome database in 2013. Nucleic Acids Res. 41, D801–D807. 10.1093/nar/gks1065 23161693 PMC3531200

[B76] Zeevi D. Korem T. Zmora N. Israeli D. Rothschild D. Weinberger A.. (2015). Personalized nutrition by prediction of glycemic responses. Cell 163, 1079–1095. 10.1016/j.cell.2015.11.001 26590418

[B77] Ziebarth J. D. Bhattacharya A. Cui Y. (2013). Bayesian network webserver: a comprehensive tool for biological network modeling. Bioinformatics 29, 2801–2803. 10.1093/bioinformatics/btt472 23969134

