# Supplementary material for: Intestinal Metagenomes and Metabolomes in Healthy Young Males: Inactivity and Hypoxia Generated Negative Physiological Symptoms Precede Microbial Dysbiosis
Source: Front Physiol. 2018 Mar 13;9:198. doi: 10.3389/fphys.2018.00198 (PMC5859311; doi:10.3389/fphys.2018.00198)
Supplement: Supplementary file 1 [file DataSheet1.pdf]

ELECTRONIC SUPPLEMENTARY MATERIAL

Intestinal metagenomes and metabolomes in healthy young males: Inactivity and hypoxia generated negative physiological symptoms precede microbial dysbiosis

Robert Šket[1], Tadej Debevec[2,3], Susanne Kublik[4], Michael Schloter[4], Anne Schoeller[4], Boštjan Murovec[5], Katarina Vogel Mikuš[6], Damjan Makuc[7], Klemen Pečnik[7], Janez Plavec[7], Igor B. Mekjavić[2], Ola Eiken[8], Zala Prevoršek[9], Blaž Stres[1,10]*

[1] Group for Microbiology and Microbial Biotechnology, Department of Animal Science, Biotechnical Faculty, University of Ljubljana, Ljubljana, Slovenia

[2] Department of Automation, Biocybernetics and Robotics, Jozef Stefan Institute, Ljubljana, Slovenia

[3]Faculty of Sport, University of Ljubljana, Ljubljana, Slovenia

[4] Research Unit for Comparative Microbiome Analysis, Helmholtz Zentrum München - German Research Center for Environmental Health, Neuherberg, Germany

[5]Machine Vision Laboratory, Faculty of Electrical Engineering, University of Ljubljana, Ljubljana, Slovenia

[6]Department of Biology, Biotechnical Faculty, University of Ljubljana, Ljubljana, Slovenia

[7]Slovenian NMR Centre, National Institute of Chemistry, Ljubljana, Slovenia

[8]Department of Environmental Physiology, Swedish Aerospace Physiology Centre, Royal Institute of Technology, Stockholm, Sweden

[9]Department of Animal Science, Biotechnical Faculty, University of Ljubljana, Ljubljana, Slovenia

[10]Center for Clinical Neurophysiology, Faculty of Medicine, University of Ljubljana, Ljubljana, Slovenia

*Corresponding author: Blaž Stres, Department of Animal Science, Biotechnical Faculty / Center for Clinical Neurophysiology, Faculty of Medicine, University of Ljubljana, Jamnikarjeva 101, 1000 Ljubljana, Slovenia. Email: blaz.stres@bf.uni-lj.si; Tel: +386 41 567 633

A)

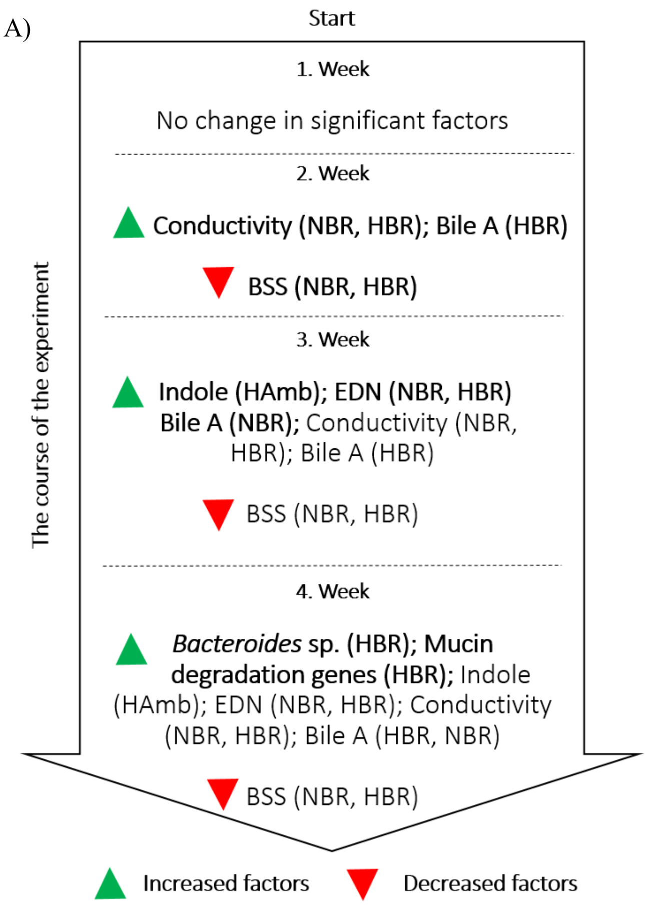

Figure S1 (continues below)

B)

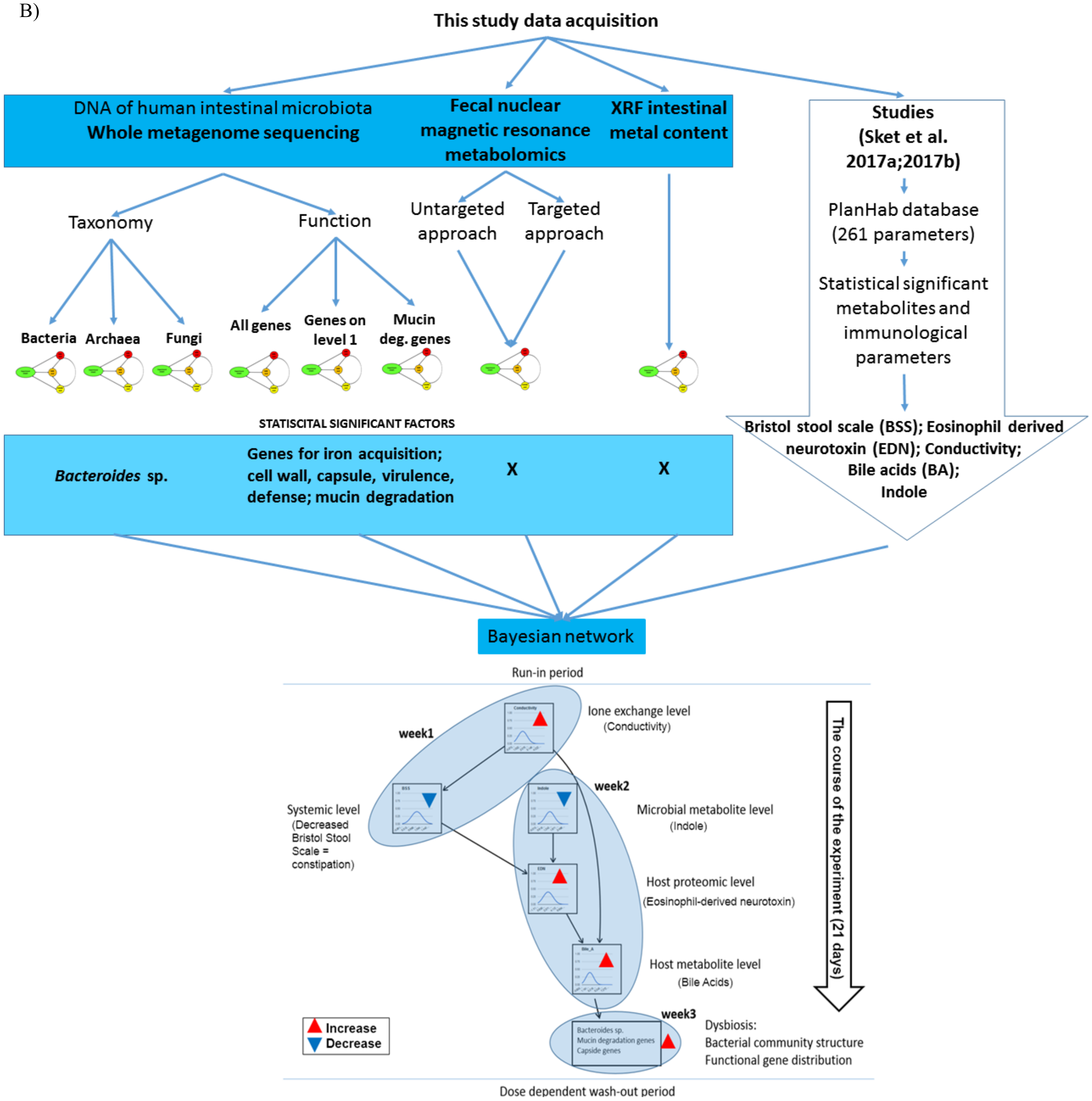

Figure S1: (A) A schematic representation of the significant changes in measured parameters of intestinal tract during the 21-day PlanHab experiments in NBR, HBR and HAmb variants reported before (Sket et al., 2017a; 2017b). **(B)** An overview of the analytical approaches adopted in this study, integrating over the different 'omics, spectroscopy, nutrition and physiology layers.

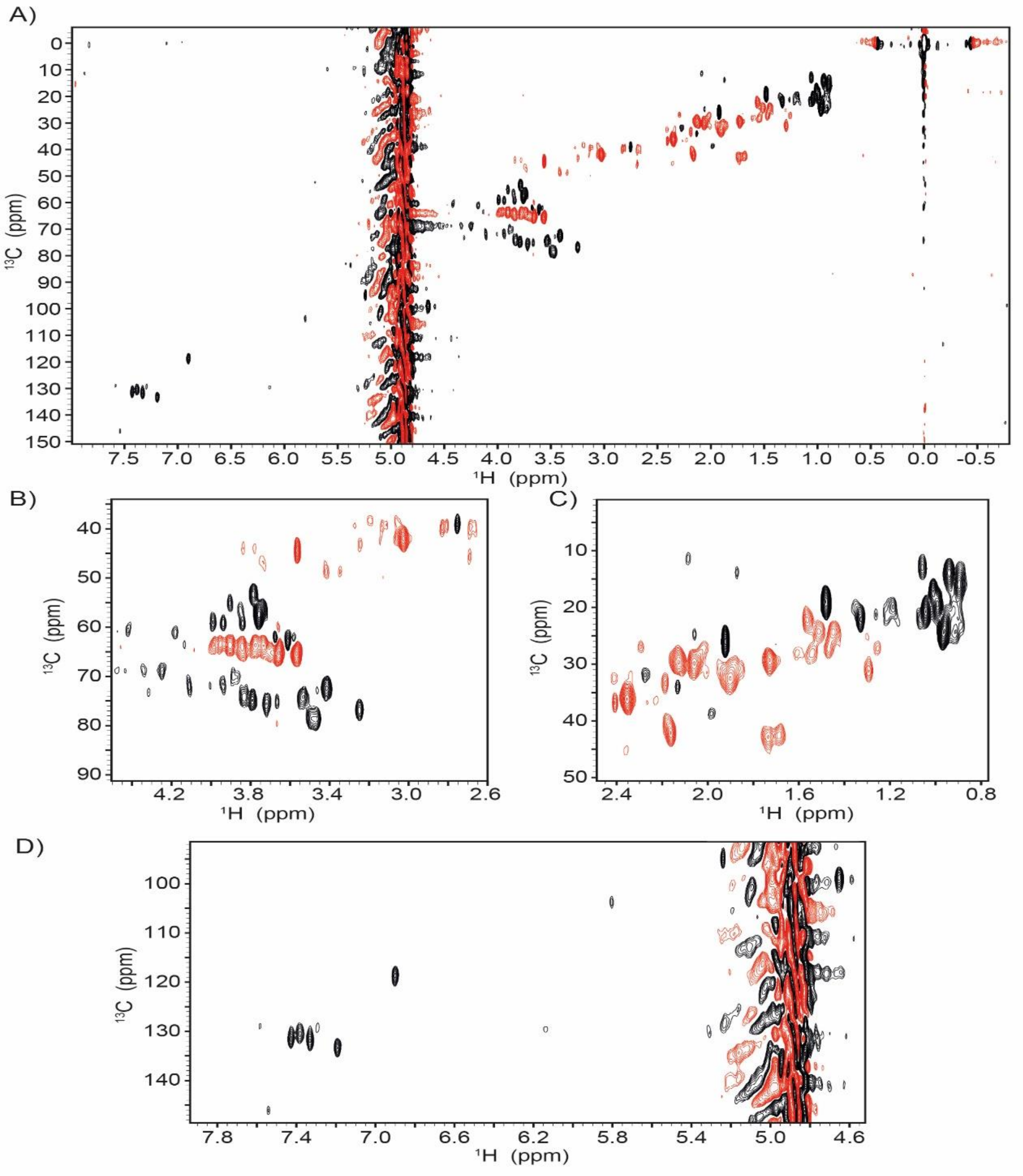

Figure S2: 2D 1H-13C NMR HSQC spectrum of sample S12_1A measured at 25 °C on 800 MHz spectrometer (A), and three zoomed sections showing aliphatic (B and C) as well as aromatic regions (D). HSQC was recorded in multiplicity-edited mode, where correlation signals in black correspond to CH and CH3 groups, whereas signals in red represent CH2 groups. Identified metabolites with assigned NMR chemical shifts are shown in Table S1.

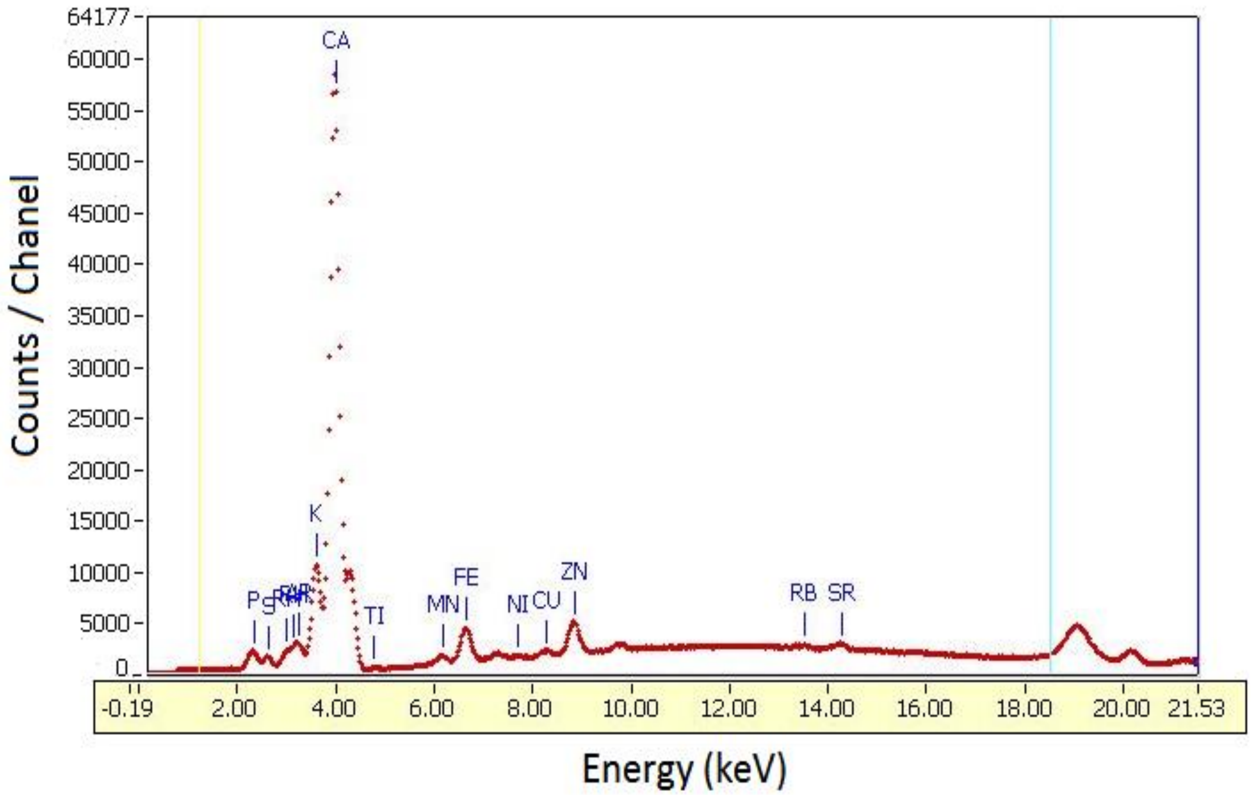

Figure S3: Fluorescence spectrum of intestinal metal content measured by X-ray fluorescence spectrometry (XRF).

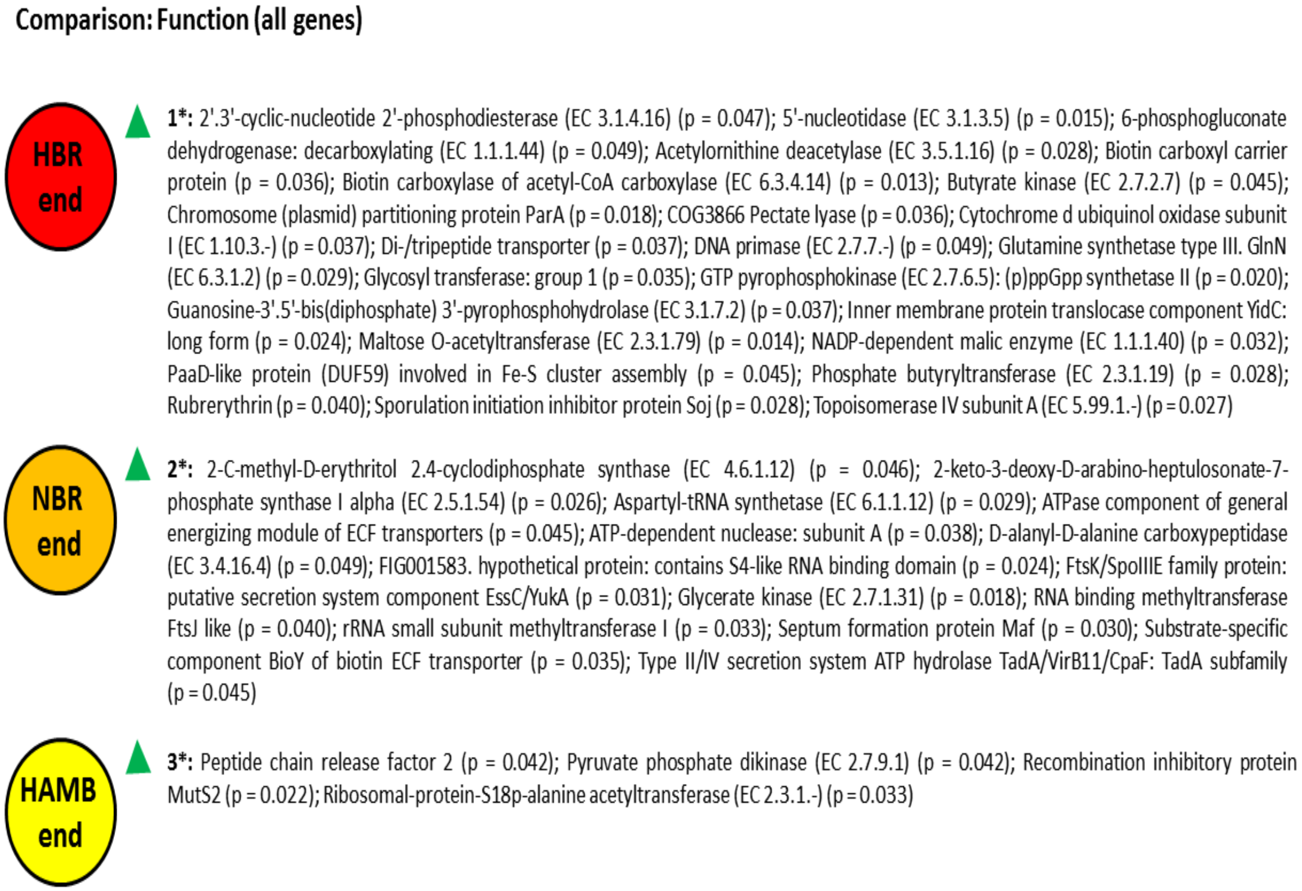

Figure S4: Statistically significantly increased genes at the functional level at the end point of NBR, HBR and HAmb variants, as described in Figure 3A.

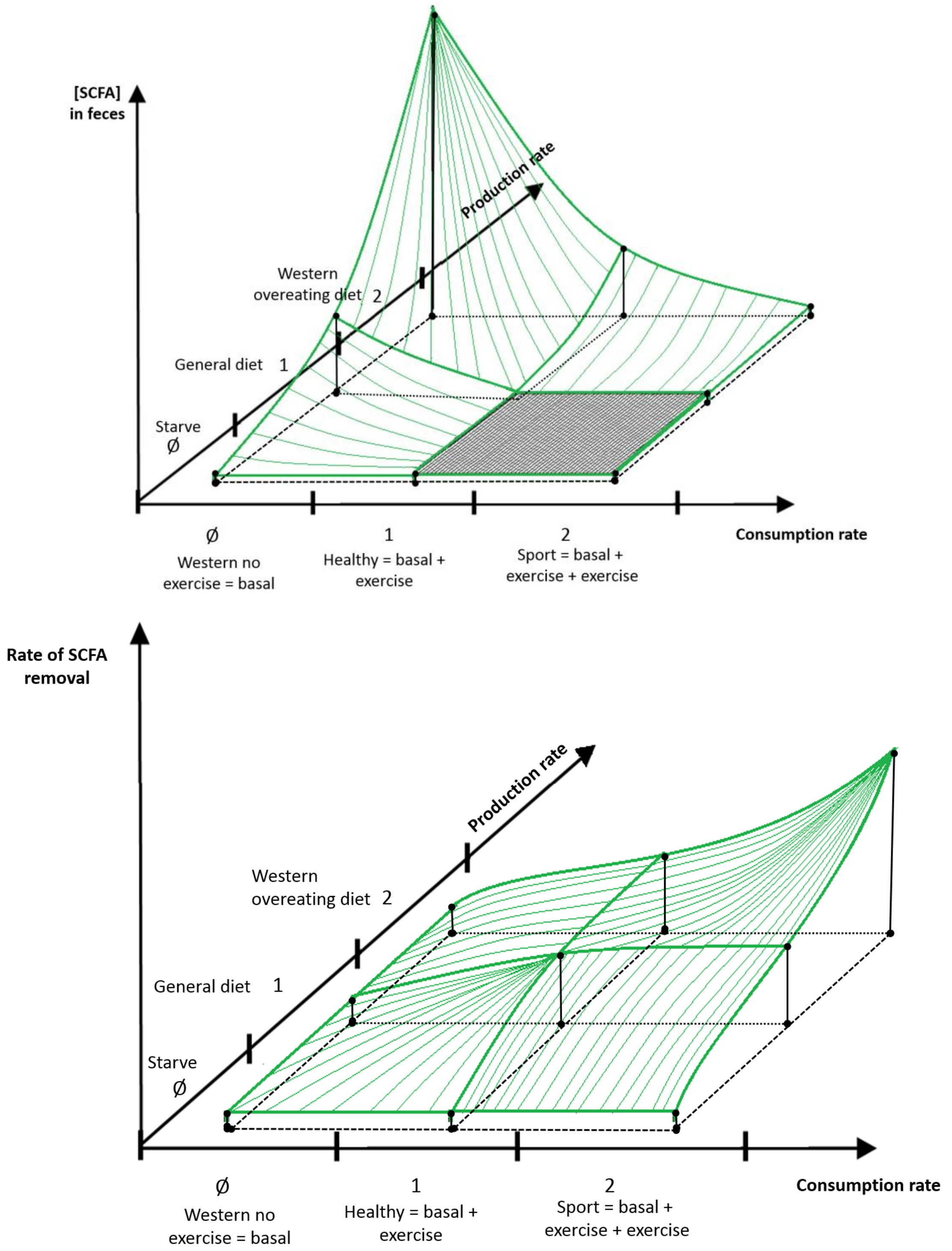

Figure S5: A conceptual framework for schematic comparison between the observed concentration of SCFA in feces (A) and the potential rates of SCFA removal into host tissue (B). These results are in line with the generally elevated SCFA concentrations in the obese population.

Table S1: ^{1}H and ^{13}C NMR chemical shifts of metabolites identified in sample S12_1A (in ppm).

Metabolite	^{1}H (ppm)	^{13}C (ppm)
A-Glucose		
	3.841	63.958
	3.777	63.684
	3.830	74.210
	3.408	72.501
	3.723	75.666
	3.533	74.244
	5.240	94.907
B-Glucose		
	3.898	63.797
	3.737	63.726
	3.471	78.744
	3.410	72.508
	3.249	76.929
	4.650	98.891
Butyric acid		
	0.898	15.947
	1.558	22.224
	2.161	42.150
Dimethylamine		
	2.754	39.197
Ethanol		
	1.187	19.925
	3.661	59.898
Glycerol		
	3.790	75.042
	3.569	65.45
	3.569	65.45
Guanidoacetic acid		
	3.740	46.541
L-Alanine		
	1.479	19.144
	3.789	53.416
L-Aspartic acid		
	3.904	55.112
	2.808	39.528
	2.685	39.627
L-Isoleucine		
	3.673	62.175

	1.985	38.711
	0.998	17.558
	1.257	27.278
	1.466	27.278
	0.942	13.934
L-Lactic acid		
	1.327	22.842
	4.111	71.939
L-Leucine		
	3.733	56.208
	1.687	42.608
	1.703	26.581
	0.961	24.805
	0.947	23.754
L-Lysine		
	3.025	42.129
	1.707	29.328
	1.449	24.312
	1.499	24.309
	1.900	32.622
	3.756	57.541
L-Phenylalanine		
	3.989	59.016
	3.125	39.274
	3.272	39.274
	7.332	131.925
	7.427	131.571
	7.383	130.531
L-Serine		
	3.837	59.388
	3.971	63.654
L-Threonine		
	3.579	62.150
	4.250	68.852
	1.327	22.085
L-Tyrosine		
	3.934	59.169
	3.192	38.496
	3.061	38.545
	7.193	133.387
	6.901	118.702

L-Valine		
	3.609	63.169
	2.274	32.088
	0.989	19.49
	1.041	20.78
L-glutamic acid		
	2.352	36.258
	2.057	30.00
	2.129	29.64
	3.758	57.524
Malonic acid		
	3.130	49.906
Pimelic acid		
	2.170	40.067
	1.540	28.132
	1.291	31.216
Propionic acid		
	1.055	12.956
	2.184	33.390
Uracil		
	5.807	103.704
	7.544	146.132
acetate		
	1.921	26.195
glycine		
	3.563	44.457

Table S2: Groups of annotated genes used for statistical analyses in Figure 3: (A) Subsystem database level 1; (B) Degradation of host mucins; (C) Butyrate synthesis pathways; (D) Aerobic and anaerobic respiration capacities.

(A) Subsystem database level 1: Amino Acids and Derivatives ; Carbohydrates ; Cell Division and Cell Cycle ; Cell Wall and Capsule ; Clustering-based subsystems ; Cofactors Vitamins Prosthetic Groups Pigments ; DNA Metabolism ; Dormancy and Sporulation ; Fatty Acids Lipids and Isoprenoids ; Iron acquisition and metabolism ; Membrane Transport ; Metabolism of Aromatic Compounds ; Miscellaneous ; Motility and Chemotaxis ; Nitrogen Metabolism ; Nucleosides and Nucleotides ; Phages Prophages Transposable elements Plasmids ; Phosphorus Metabolism ; Photosynthesis ; Potassium metabolism ; Protein Metabolism ; Regulation and Cell signaling ; Respiration ; RNA Metabolism ; Secondary Metabolism ; Stress Response ; Sulfur Metabolism ; Virulence Disease and Defense.

(B) Degradation of host mucins: Sialidase (EC 3.2.1.18); Beta-galactosidase (EC 3.2.1.23); Beta-galactosidase (EC 3.2.1.23) LacA family; Beta-galactosidase (EC 3.2.1.23) LacZ family; Beta-galactosidase 3; Beta-galactosidase large subunit (EC 3.2.1.23); Beta-galactosidase small subunit (EC 3.2.1.23); Alpha-N-acetylglucosaminidase (EC 3.2.1.50); Alpha-L-fucosidase (EC 3.2.1.51); Beta-hexosaminidase (EC 3.2.1.52); Alpha-galactosidase (EC 3.2.1.22); Alpha-galactosidase precursor (EC 3.2.1.22).

(C) Butyrate synthesis pathways: Butyrate kinase (EC 2.7.2.7); Butyrate-acetoacetate CoA-transferase subunit A (EC 2.8.3.9); Butyrate-acetoacetate CoA-transferase subunit B (EC 2.8.3.9); Butyryl-CoA dehydrogenase (EC 1.3.99.2); D-beta-hydroxybutyrate dehydrogenase (EC 1.1.1.30); D-beta-hydroxybutyrate permease; 3-hydroxybutyryl-CoA dehydratase (EC 4.2.1.55); 3-hydroxybutyryl-CoA dehydrogenase (EC 1.1.1.157); 2-amino-3-ketobutyrate coenzyme A ligase (EC 2.3.1.29); Acetyl-CoAacetoacetyl-CoA transferase. alpha subunit (EC 2.8.3.8); Lysine 2.3-aminomutase (EC 5.4.3.2); L-beta-lysine 5.6-aminomutase alpha subunit (EC 5.4.3.3); L-beta-lysine 5.6-aminomutase beta subunit (EC 5.4.3.3); 3-keto-5-aminohexanoate cleavage enzyme; 3-aminobutyrl-CoA ammonia lyase (EC 4.3.1.14); Phosphate butyryltransferase (EC 2.3.1.19) ;3-ketoacyl-CoA thiolase (EC 2.3.1.16).

(D) Aerobic and anaerobic respiration capacities: 2.4-dienoyl-CoA reductase (NADPH) (EC 1.3.1.34); 2-dehydropantoate 2-reductase (EC 1.1.1.169); 2-hydroxy-3-oxopropionate reductase (EC 1.1.1.60); 2-polyprenylphenol hydroxylase and related flavodoxin oxidoreductases; 3-oxoacyl-(acyl-carrier protein) reductase (EC 1.1.1.100); 3-oxoacyl-(acyl-carrier protein) reductase paralog (EC 1.1.1.100) in cluster with unspecified monosaccharide transporter; 4-hydroxy-3-methylbut-2-enyl diphosphate reductase (EC 1.17.1.2); 5.10-methylenetetrahydrofolate reductase (EC 1.5.1.20); 5-amino-6-(5-phosphoribosylamino)uracil reductase (EC 1.1.1.193); 5-keto-D-gluconate 5-reductase (EC 1.1.1.69); Acetoacetyl-CoA reductase (EC 1.1.1.36); Adenylylsulfate reductase alpha-subunit (EC 1.8.99.2); Adenylylsulfate reductase beta-subunit (EC 1.8.99.2); Aldo-keto reductase family 1 member B10 (EC 1.1.1.-); Alkyl hydroperoxide reductase protein C (EC 1.6.4.-); Alkyl hydroperoxide reductase protein F (EC 1.6.4.-); Alkyl hydroperoxide reductase subunit C-like protein; Altronate oxidoreductase (EC 1.1.1.58); Anaerobic dimethyl sulfoxide reductase chain A (EC 1.8.99.-); Anaerobic dimethyl sulfoxide reductase chain B (EC 1.8.99.-); Arsenate reductase (EC 1.20.4.1); Benzoyl-CoA reductase subunit BadE (EC 1.3.99.15); Benzoyl-CoA reductase subunit BadF (EC 1.3.99.15); Benzoyl-CoA reductase subunit BadG (EC 1.3.99.15); CoA-disulfide reductase (EC 1.8.1.14); Cob(II)alamin reductase; Cob(III)alamin reductase; Cobalt-precorrin-6x reductase (EC 1.3.1.54); CoB--CoM heterodisulfide reductase subunit A (EC 1.8.98.1); CoB--CoM heterodisulfide reductase subunit B (EC 1.8.98.1); CoB--CoM heterodisulfide reductase subunit C (EC 1.8.98.1); CoB--CoM heterodisulfide reductase subunit D (EC 1.8.98.1); Coenzyme A disulfide reductase; Cytochrome c nitrite reductase. small subunit NrfH; Cytochrome c-type biogenesis protein DsbD. protein-disulfide reductase (EC 1.8.1.8); Dihydrodipicolinate reductase (EC 1.3.1.26); Dihydroflavonol-4-reductase (EC 1.1.1.219); Dihydrofolate reductase (EC 1.5.1.3); Dissimilatory sulfite reductase (desulfoviridin). alpha and beta subunits; D-mannonate oxidoreductase (EC 1.1.1.57); D-proline reductase. 23 kDa subunit (EC 1.21.4.1); D-proline reductase. 45 kDa subunit (EC 1.21.4.1); dTDP-4-dehydrorhamnose reductase (EC 1.1.1.133); Enoyl-(acyl-carrier-protein) reductase (FMN) (EC 1.3.1.9); Enoyl-(acyl-carrier-protein) reductase (NADH) (EC 1.3.1.9); Ferredoxin reductase; Ferredoxin-type protein NapG (periplasmic nitrate reductase); Fumarate reductase flavoprotein subunit (EC 1.3.99.1); Gamma-glutamyl phosphate reductase (EC 1.2.1.41); Glutamyl-tRNA reductase (EC 1.2.1.70); Glycine reductase component B alpha subunit (EC 1.21.4.2); Glycine reductase component B beta subunit (EC 1.21.4.2); Glycine reductase component B gamma subunit (EC 1.21.4.2);

Glycine/sarcosine/betaine reductase component C chain 1; Glycine/sarcosine/betaine reductase component C chain 2; Glyoxylate reductase (EC 1.1.1.26); Glyoxylate reductase (EC 1.1.1.79); Heterodisulfide reductase. cytochrome reductase subunit; heterodisulfide reductase. iron-sulfur binding subunit. putative; heterodisulfide reductase. subunit A/methylviologen reducing hydrogenase. subunit delta; Hydroxylamine reductase (EC 1.7.-.-); Hydroxymethylglutaryl-CoA reductase (EC 1.1.1.34); Hydroxypyruvate reductase (EC 1.1.1.81); Hypothetical oxidoreductase YdjG (EC 1.-.-.-); Indolepyruvate oxidoreductase subunit IorA (EC 1.2.7.8); Indolepyruvate oxidoreductase subunit IorB (EC 1.2.7.8); Ketoisovalerate oxidoreductase subunit VorA (EC 1.2.7.7); Ketoisovalerate oxidoreductase subunit VorB (EC 1.2.7.7); Lactaldehyde reductase (EC 1.1.1.77); L-sorbose 1-phosphate reductase (EC 1.1.1.-); Mercuric ion reductase (EC 1.16.1.1); Na(+)-translocating NADH-quinone reductase subunit A (EC 1.6.5.-); Na(+)-translocating NADH-quinone reductase subunit B (EC 1.6.5.-); Na(+)-translocating NADH-quinone reductase subunit C (EC 1.6.5.-); Na(+)-translocating NADH-quinone reductase subunit D (EC 1.6.5.-); Na(+)-translocating NADH-quinone reductase subunit E (EC 1.6.5.-); Na(+)-translocating NADH-quinone reductase subunit F (EC 1.6.5.-); N-acetyl-gamma-glutamyl-phosphate reductase (EC 1.2.1.38); NAD(P)H oxidoreductase YRKL (EC 1.6.99.-); NADH ubiquinone oxidoreductase chain A (EC 1.6.5.3); NADH-ubiquinone oxidoreductase chain B (EC 1.6.5.3); NADH-ubiquinone oxidoreductase chain C (EC 1.6.5.3); NADH-ubiquinone oxidoreductase chain D (EC 1.6.5.3); NADH-ubiquinone oxidoreductase chain E (EC 1.6.5.3); NADH-ubiquinone oxidoreductase chain F (EC 1.6.5.3); NADH-ubiquinone oxidoreductase chain H (EC 1.6.5.3); NADH-ubiquinone oxidoreductase chain I (EC 1.6.5.3); NADH-ubiquinone oxidoreductase chain J (EC 1.6.5.3); NADH-ubiquinone oxidoreductase chain K (EC 1.6.5.3); NADH-ubiquinone oxidoreductase chain L (EC 1.6.5.3); NADH-ubiquinone oxidoreductase chain M (EC 1.6.5.3); NADH-ubiquinone oxidoreductase chain N (EC 1.6.5.3); NADPH-dependent 7-cyano-7-deazaguanine reductase (EC 1.7.1.-); Nitric oxide reductase activation protein NorD; Nitric oxide reductase activation protein NorQ; Nitrite reductase (NAD(P)H) large subunit (EC 1.7.1.4); Nitrite reductase probable (NAD(P)H) subunit (EC 1.7.1.4); Nitrite reductase probable electron transfer 4Fe-S subunit (EC 1.7.1.4); Nitrogenase (molybdenum-iron) reductase and maturation protein NifH; Peptide methionine sulfoxide reductase MsrA (EC 1.8.4.11); Peptide methionine sulfoxide reductase MsrB (EC 1.8.4.12); PF00070 family. FAD-dependent NAD(P)-disulphide oxidoreductase; Polyferredoxin NapH (periplasmic nitrate reductase); Predicted L-lactate dehydrogenase. Fe-S

oxidoreductase subunit YkgE; Probable electron transfer flavoprotein-quinone oxidoreductase FixC (EC 1.5.5.-); Probable thiol oxidoreductase with 2 cytochrome c heme-binding sites; PUA-PAPS reductase like fusion; Putative oxidoreductase linked to yggC; Putative oxidoreductase YdjL; Pyrroline-5-carboxylate reductase (EC 1.5.1.2); Pyruvateferredoxin oxidoreductase. alpha subunit (EC 1.2.7.1); Pyruvateferredoxin oxidoreductase. beta subunit (EC 1.2.7.1); Pyruvateferredoxin oxidoreductase. delta subunit (EC 1.2.7.1); Pyruvateferredoxin oxidoreductase. gamma subunit (EC 1.2.7.1); Pyruvate-flavodoxin oxidoreductase (EC 1.2.7.-); Respiratory nitrate reductase alpha chain (EC 1.7.99.4); Ribonucleotide reductase of class Ia (aerobic). alpha subunit (EC 1.17.4.1); Ribonucleotide reductase of class Ia (aerobic). beta subunit (EC 1.17.4.1); Ribonucleotide reductase of class Ib (aerobic). alpha subunit (EC 1.17.4.1); Ribonucleotide reductase of class Ib (aerobic). beta subunit (EC 1.17.4.1); Ribonucleotide reductase of class II (coenzyme B12-dependent) (EC 1.17.4.1); Ribonucleotide reductase of class III (anaerobic). activating protein (EC 1.97.1.4); Ribonucleotide reductase of class III (anaerobic). large subunit (EC 1.17.4.2); Ribonucleotide reductase transcriptional regulator NrdR; Superoxide reductase (EC 1.15.1.2); Thioldisulfide oxidoreductase related to ResA; Thioredoxin reductase (EC 1.8.1.9); Trimethylamine-N-oxide reductase (EC 1.6.6.9); UDP-N-acetylenolpyruvoylglucosamine reductase (EC 1.1.1.158)
